# Gut Microbiota Colonization in Early Life Influences Susceptibility to Adulthood Hepatic Lipid Accumulation in High‐Fat‐Diet‐Fed Mice

**DOI:** 10.1002/advs.202412827

**Published:** 2025-04-07

**Authors:** Yan‐Yan Zhu, Xin Dong, Hao Zhou, Ze‐Yan Li, Bo Wang, Ya‐Ping Song, Zhi‐Bing Liu, Xue Lu, Yi‐Hao Zhang, Yichao Huang, Hua Wang, De‐Xiang Xu

**Affiliations:** ^1^ Department of Toxicology School of Public Health Anhui Medical University Hefei 230032 China; ^2^ Key Laboratory of Environmental Toxicology of Anhui Higher Education Institutes Anhui Medical University Hefei 230032 China; ^3^ Department of Blood Transfusion the Second Affiliated Hospital of Anhui Medical University Hefei 230601 China

**Keywords:** amoxicillin, bile acid metabolism, gut microbiota colonization, lactobacillus supplementation, metabolic dysfunction‐associated fatty liver disease

## Abstract

The global prevalence of Metabolic Dysfunction‐Associated Fatty Liver Disease (MAFLD) has a rising trend. The Developmental Origins of Health and Disease (DOHaD) theory assumes that MAFLD develops throughout the entire lifecycle but it originates in early life. This study aimed to investigate the influence of early‐life gut microbiota colonization on the susceptibility to adulthood hepatic lipid accumulation in high‐fat‐diet (HFD)‐fed mice. The results showed that perinatal AM exposure exacerbated adulthood hepatic lipid accumulation and altered hepatic lipid profile in HFD‐fed male but not female offspring. Perinatal AM exposure does not affect hepatic lipid metabolic genes in adult offspring. Instead, perinatal AM exposure inhibited intestinal bile acid (BA) metabolism to reduce secondary BAs production, thereby promoting dietary lipid absorption. Mechanistically, perinatal AM exposure permanently reduces species diversity of the microbial community and impairs its structure and function by disrupting early‐life gut microbiota colonization. Supplementing Lactobacillus during lactation improved gut microbiota colonization and intestinal BA metabolism, thereby alleviating HFD‐induced hepatic lipid deposition. These results suggest that disruption of early‐life gut microbiota colonization elevates susceptibility to adulthood hepatic lipid accumulation by promoting intestinal lipid absorption in HFD‐fed mice. Supplementing probiotics during lactation may be an effective strategy for preventing susceptibility to adulthood MAFLD.

## Graphical abstract

This study indicates that perinatal antibiotic exposure elevates susceptibility to high‐fat‐diet‐induced hepatic lipid accumulation via disrupting early‐life gut microbiota colonization. The decreased Lactobacillus colonization inhibits primary‐to‐secondary bile acid conversion, lowering secondary bile acids, enhancing adulthood intestinal lipid absorption and hepatic lipid deposition. Supplementing with Lactobacillus during lactation improves early‐life gut microbiota colonization and alleviates high‐fat‐diet‐induced hepatic lipid accumulation in adulthood.

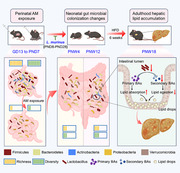

## 1 Introduction

The Metabolic Dysfunction‐Associated Fatty Liver Disease (MAFLD) refers to a chronic liver disorder characterized by hepatic lipid deposition and accompanied by overweight or obesity, type 2 diabetes, and metabolic dysregulation.^[^ ^1^, ^2^ ^]^ It is estimated that MAFLD influences more than one‐third of the world's population and has become one of the most important public health issues.^[^ ^3^, ^4^ ^]^ It is accepted that a sedentary lifestyle, high‐sugar or high‐fat diets, advanced age, and hyperlipidemia are all risk factors for MAFLD.^[^ ^5^, ^6^, ^7^ ^]^ The abnormal hepatic lipid accumulation plays a central role in the development of MAFLD.^[^ ^8^, ^9^ ^]^ Recently, the Developmental Origins of Health and Disease theory assumes that MAFLD develops throughout the entire lifecycle but it originates in early life.^[^ ^10^, ^11^ ^]^ Increasing data have demonstrated that exposure to adverse environments in early life predisposes to adulthood MAFLD.^[^ ^12^, ^13^, ^14^ ^]^ Nevertheless, the exact mechanisms need to be determined.

Numerous studies indicate that alterations in epigenetic and metabolic reprogramming are the key mechanism for the susceptibility to adulthood MAFLD.^[^ ^15^, ^16^, ^17^, ^18^ ^]^ Recently, two reports demonstrated that early‐life exposure to toxicant causes adulthood steatosis by altering hydroxymethylation reprogramming of hepatic β‐oxidation genes.^[^ ^19^, ^20^ ^]^ On the other hand, increasing evidences have suggested a specific association between gut microbiota dysbiosis and MAFLD.^[^ ^21^, ^22^, ^23^ ^]^ Gut microbiota colonization refers to the process by which microbial communities within the host's intestines evolve from an initial sterile or low‐diversity state, through a series of complex ecological processes, to gradually establish, succeed, and ultimately form a relatively stable and diverse microbial ecosystem.^[^ ^24^ ^]^ It is known that gut microbiota is colonized in early life.^[^ ^25^, ^26^ ^]^ Several studies demonstrate that gut microbiota colonization in early life impacts health throughout the entire lifecycle.^[^ ^27^, ^28^, ^29^ ^]^ In this study, we hypothesize that adverse factors in early life increase the susceptibility to adulthood MAFLD by influencing gut microbiota colonization in early life.

Amoxicillin (AM) is a commonly used antibiotic during the perinatal period, typically used for treating premature rupture of membranes to reduce the incidence of maternal and neonatal infections.^[^ ^30^, ^31^ ^]^ In this study, we aimed to construct a mouse model of early‐life gut microbiota colonization changes using perinatal AM exposure. The impacts of early‐life gut microbiota colonization on the susceptibility of high‐fat‐diet (HFD)‐induced MAFLD were then investigated. Our results showed that perinatal AM exposure altered the colonization of gut microbiota in early life and elevated the susceptibility to HFD‐induced hepatic lipid deposition in adulthood. We provide evidence that early‐life gut microbiota colonization impacts the susceptibility to adulthood hepatic lipid accumulation by disrupting intestinal bile acid (BA) metabolism and dietary lipid absorption.

## 2 Results

### 2.1 Perinatal AM Exposure Exacerbates Hepatic Lipid Deposition in HFD‐Fed Male Offspring

**Figure 1:**
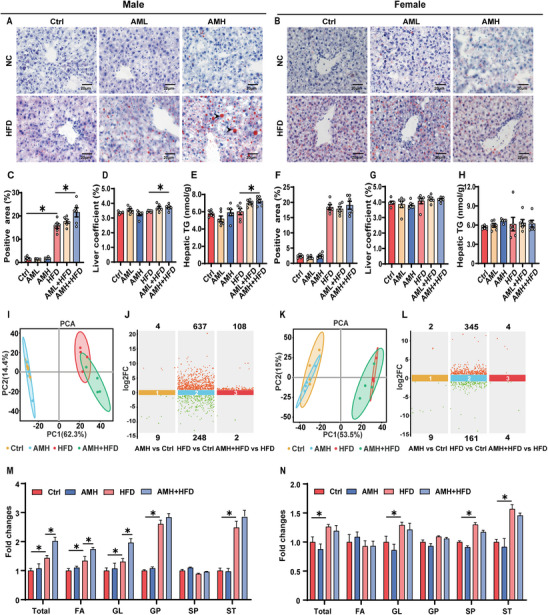
Impacts of perinatal AM exposure on hepatic lipid accumulation and lipid profile in HFD‐fed adult offspring. In animal experiment 1, hepatic tissues were collected at PNW19. A–C,F) Oil Red O staining was performed on liver sections (×400 magnification) for quantitative analysis. D,E,G,H) Liver coefficients and hepatic TG content were also measured (*n* = 5–6). I–N) A lipidome analysis provided a hepatic lipid profile. I,K) PCA score plots. J,L) Dynamic differential scatter plots. M,N) Quantitative analyses for lipid subclass abundance were conducted (*n* = 4). All data are expressed as mean ± SEM. Statistical significance was determined using two‐sided one‐way ANOVA with post hoc LSD tests. Significance was set at ^*^ *p* < 0.05.

Before HFD feeding, body weight changes were compared. As presented in Figure S1A,C (Supporting Information), no discrepancy was noted among the various groups. At 6 weeks after HFD feeding, body weight was increased in AMH‐exposed male pups (Figure S1B, Supporting Information), whereas no difference was observed between AMH‐exposed female offspring and controls (Figure S1D, Supporting Information). Food intake was measured and showed no difference among different groups (Figure S1E,G, Supporting Information). Brown adipose tissue (BAT), subcutaneous white adipose tissue (sWAT), perirenal white adipose tissue (pWAT), and gonadal white adipose tissue (gWAT) were measured. As shown in Figure S1F (Supporting Information), gWAT was increased in AMH‐exposed HFD‐fed male offspring, with no difference in other tissues (Figure S1F,H, Supporting Information). ALT was elevated in male and female offspring (Figure S1I,L, Supporting Information), but AST was elevated only in male offspring (Figure S1J,M, Supporting Information). As shown in Figure S1N–T (Supporting Information), no difference was observed in plasma TC, LDL, and HDL. Interestingly, plasma TG was higher in AM‐exposed HFD‐fed male offspring than in HFD‐fed male offspring (Figure S1K, Supporting Information). Next, hepatic lipids were evaluated. H&E staining showed that NAFLD activity score was elevated in AMH‐exposed HFD‐fed male (Figure S2A,C, Supporting Information) and female offspring (Figure S2B,E, Supporting Information). Oil Red‐O staining revealed more hepatic lipid droplets in AMH‐exposed HFD‐fed male offspring than in HFD‐fed male offspring (**Figure** **1**A,C). No difference on hepatic lipid droplets was observed between AMH‐exposed HFD‐fed female offspring and HFD‐fed female offspring (Figure 1B,F). Liver weight and coefficient were analyzed and showed increased in AMH‐exposed HFD‐fed male (Figures S2D and 1D, Supporting Information) but not female offspring (Figures S2F and 1G, Supporting Information). Finally, hepatic TG content was measured and showed increased in AMH‐exposed HFD‐fed male but not female offspring (Figure 1E,H).

### 2.2 Perinatal AM Exposure Alters Hepatic Lipid Profiles in HFD‐Fed Male Offspring

Lipidome was performed to analyze hepatic lipid profile. The OPLS‐DA score map (Figure 1I,K) represented differential lipid metabolites among different groups. Hepatic lipid profiles in AMH‐exposed HFD‐fed male offspring were different from those in HFD‐fed male offspring (Figure 1I), whereas no difference was observed in AMH‐exposed HFD‐fed female offspring compared with HFD‐fed female offspring (Figure 1K). The volcano plot was used to analyze the differential lipid metabolites (Figure 1J,L). There were four lipids increased and nine lipids decreased in AMH‐exposed male offspring. Moreover, 637 lipids were increased and 248 lipids were decreased in HFD‐fed male offspring. Interestingly, 108 lipids were elevated and only two lipids were reduced in AMH‐exposed HFD‐fed male offspring compared to HFD‐fed male offspring (Figure 1J). There were only two lipids increased and nine lipids decreased in AMH‐exposed female offspring. Moreover, 345 lipids were increased and 248 lipids were decreased in HFD‐fed female offspring. Only four lipids were increased and four lipids were decreased in AMH‐exposed HFD‐fed female offspring compared to HFD‐fed female offspring (Figure 1L). Lipids are categorized into eight primary categories: fatty acyls (FA), glycerolipids (GL), glycerophospholipids (GP), sphingolipids (SP), sterol lipids (ST), prenol lipids (PR), saccharolipids (SL), and polyketides (PK). Elevated glycerolipids are one of the primary characteristics of hepatic lipid deposition.^[^ ^32^ ^]^ Detailed lipidomic analysis revealed that total lipids, FA, GL, GP, and ST were elevated in HFD‐fed male offspring. The heat maps displayed obvious differences among different groups (Figure S2G,H, Supporting Information). HFD‐evoked elevation of total lipids, FA, and GL was aggravated in AMH‐exposed HFD‐fed male offspring (Figure 1M). Although total lipids, GL, SP, and ST were elevated, perinatal AM exposure did not exacerbate HFD‐evoked elevation of hepatic lipids in female offspring (Figure 1N).

### 2.3 Perinatal AM Exposure does not Affect Basal Metabolism in Adult Offspring

Food intake within 48 h was evaluated. For males, no variation in food intake was noted across the different groups (Figure S3A–C, Supporting Information). Food intake within 48 h was reduced in AM‐exposed females (Figure S3D,F, Supporting Information). Moreover, food intake was reduced in AM‐exposed HFD‐fed females compared with HFD‐fed females (Figure S3E,F, Supporting Information). Respiratory exchange rate (RER) was used to evaluate basal metabolism. In this experiment, an RER close to 1 indicates that the body mainly consumed carbohydrates and an RER close to 0.7 indicates that the body primarily used fat for energy. For males (Figure S3G‐I, Supporting Information) and females (Figure S3J–L, Supporting Information), no difference on RER values within 48 h was shown among different groups. Next, locomotor activity within 48 h was monitored. For males (Figure S3M–O, Supporting Information) and females (Figure S3P–R, Supporting Information), there was no difference on locomotor activity within 48 h among different groups. Finally, energy expenditure (EE) was compared among different groups. For males (Figure S3S–U, Supporting Information) and females (Figure S3V–X, Supporting Information), there was no difference on energy expenditure within 48 h among different groups.

### 2.4 Perinatal AM Exposure does not Alter Hepatic Lipid Metabolic Gene Expression in Adult Offspring

Transcriptomics was performed to analyze hepatic lipid metabolism‐related genes (Figure S4A,C, Supporting Information). Dynamic multi‐group differential scatter plots were used to analyze differential gene expression. As shown in Figure S4B (Supporting Information), there were 76 genes upregulated and 52 genes downregulated in AMH‐exposed male offspring. Moreover, 315 upregulated genes and 316 downregulated genes in HFD‐fed male offspring. Compared to HFD‐fed males, 91 upregulated genes and 42 downregulated genes in AMH‐exposed HFD‐fed males (Figure S4B, Supporting Information). For AMH‐exposed female offspring, 70 upregulated genes and 24 downregulated genes. Moreover, 92 upregulated genes and 50 downregulated genes in HFD‐fed female offspring. Only 25 upregulated genes and 22 downregulated genes in AMH‐exposed HFD‐fed female offspring compared with HFD‐fed females (Figure S4D, Supporting Information). Functional annotation and enrichment analysis were performed using GO and KEGG enrichment analysis. (Figure S4E–L, Supporting Information). In the differential gene GO pathway enrichment analysis, no lipid metabolic pathways were found to be enriched. Similarly, in the differential gene KEGG pathway enrichment analysis, no pathways related to lipid metabolism were enriched. Finally, real‐time RT‐PCR was used to detect hepatic lipid metabolism‐related genes. No difference on hepatic lipid metabolism‐related genes, including lipid synthesis, fatty acid binding proteins, β‐oxidation, lipid degradation, and lipid transport, was shown among different groups (Figure S4M,N, Supporting Information).

### 2.5 AM Exposure Enhances Intestinal Lipid Absorption in Male Mice

**Figure 2:**
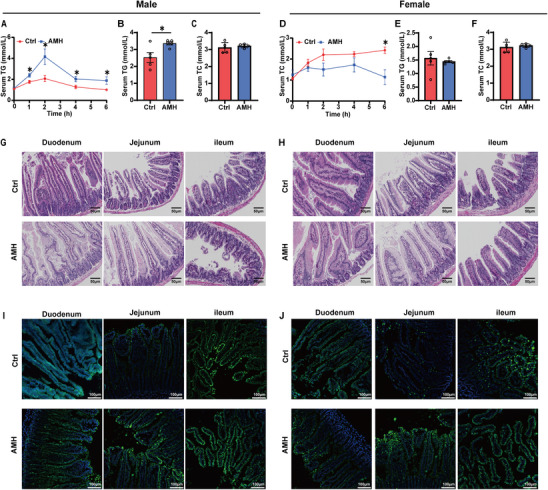
Impacts of AM exposure on intestinal lipid absorption. A,D) In animal experiment 2, serum triglyceride levels were measured at 0, 1, 2, 4, and 6 h after olive oil gavage (*n* = 4–6). In experiment 3, serum and small intestine tissue were collected to assess serum triglyceride (TG) and total cholesterol (TC) content and perform H&E staining of intestine sections (*n* = 5). I,J) In experiment 4, small intestine tissue was collected for fluorescence imaging (*n* = 4–6). Data are presented as mean ± SEM. Statistical significance was determined by two‐way ANOVA with post hoc LSD tests for (A,D), and two‐tailed Student's *t*‐test for (B–F), with ^*^ *p* < 0.05 indicating significance.

Postprandial TG response was performed to test intestinal lipid absorption. As shown in **Figure** **2**A, serum TG content was elevated at all time points after AM‐exposed males were administered with olive oil. No difference on serum TG was observed between AM‐exposed females and controls. Serum TG content was reduced at 6 h after AM‐exposed females were administered with olive oil (Figure 2D). Next, the serum and intestinal lipids were measured in mice that had been fasted overnight and then refed with a 60% HFD for 2 h. Serum TG content was increased in AM‐exposed males (Figure 2B) but not in AM‐exposed females (Figure 2E). Serum TC wasn't elevated in AM‐exposed males and females (Figure 2C,F). Histology revealed that enterocytes in duodenum and jejunum contained a large amount of lipid droplets at 2 h after AM‐exposed males were fasted overnight and refed with 60% HFD (Figure 2G). Only a slight elevation of lipid droplets was shown in AM‐exposed HFD‐fed females (Figure 2H). Finally, BODIPY‐labeled FA was administered to directly visualize FA uptake into enterocytes. Abundant fluorescence‐labeled lipid droplets were shown in intestines of AM‐exposed males (Figure 2I) but not females (Figure 2J).

### 2.6 Perinatal AM Exposure Disrupts Fecal BA Composition in Male Offspring

**Figure 3:**
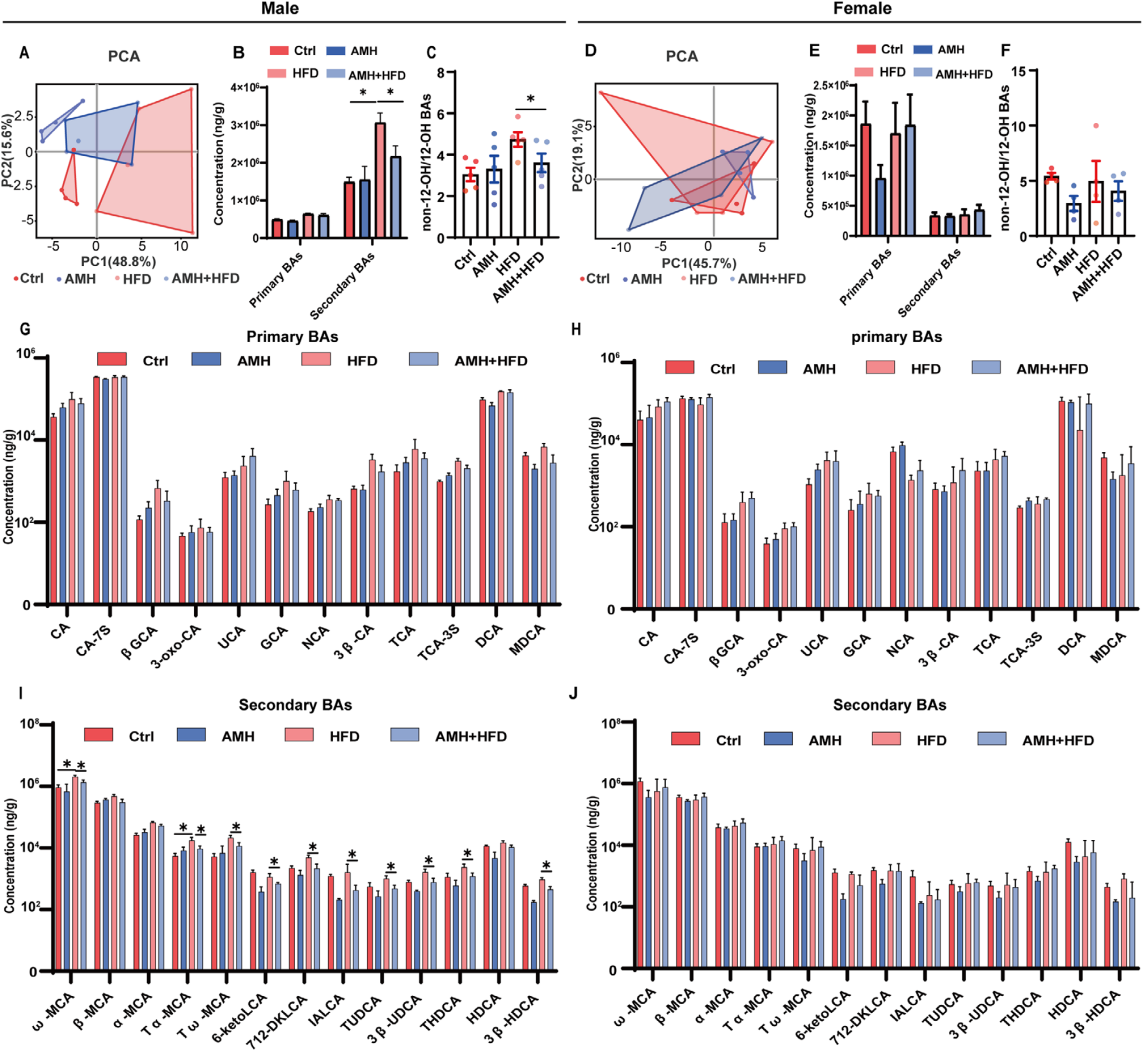
Impacts of perinatal AM exposure on fecal BA composition in HFD‐fed adult offspring. In animal experiment 1, Cecum contents were collected at PNW 19 to test BAs. A,D) PCA score plots. B,E) Primary and secondary BAs. C,F) Ratio of non‐12‐OH to 12‐OH BAs. G,H) Primary BA concentrations. I,J) Secondary BA concentrations. Data are expressed as mean ± SEM; significance was assessed with two‐sided one‐way ANOVA and post hoc LSD tests. *n* = 4–5. ^*^ *p* < 0.05.

Fecal BA composition was determined by UPLC‐MS. Principal component analysis of fecal BA profiles showed an obvious separation between AM‐exposed male offspring and controls (**Figure** **3**A) but not between AM‐exposed female offspring and controls (Figure 3D). An obvious separation was observed between AM‐exposed HFD‐fed male offspring and HFD‐fed male offspring (Figure 3A) but not between AM‐exposed HFD‐fed female offspring and HFD‐fed female offspring (Figure 3D). Despite no change on primary BAs, secondary BAs were increased in HFD‐fed male offspring (Figure 3B) but not in HFD‐fed female offspring (Figure 3E). Accordingly, the ratio of non‐12‐OH BAs to 12‐OH BAs was increased in HFD‐fed male offspring (Figure 3C) but not in HFD‐fed female offspring (Figure 3F). Of interest, perinatal AM exposure attenuated HFD‐induced elevation of secondary BAs in male offspring (Figure 3B). Moreover, perinatal AM exposure attenuated increase in the ratio of non‐12‐OH BAs to 12‐OH BAs in HFD‐fed male offspring (Figure 3C). The impact of perinatal AM exposure on fecal BA composition was further analyzed. Perinatal AM exposure had little effect on primary BAs in males and females (Figure 3G,H). Secondary BAs, including ω‐MCA, Tα‐MCA, Tω‐MCA, 6‐ketoLCA, IALCA, TUDCA, 3β‐UDCA, THDCA, and 3β‐HDCA, were reduced in AM‐exposed HFD‐fed male (Figure 3I) but not in female offspring (Figure 3J).

### 2.7 Perinatal AM Exposure Disrupts Composition of Gut Microbiota in Adult Offspring

**Figure 4:**
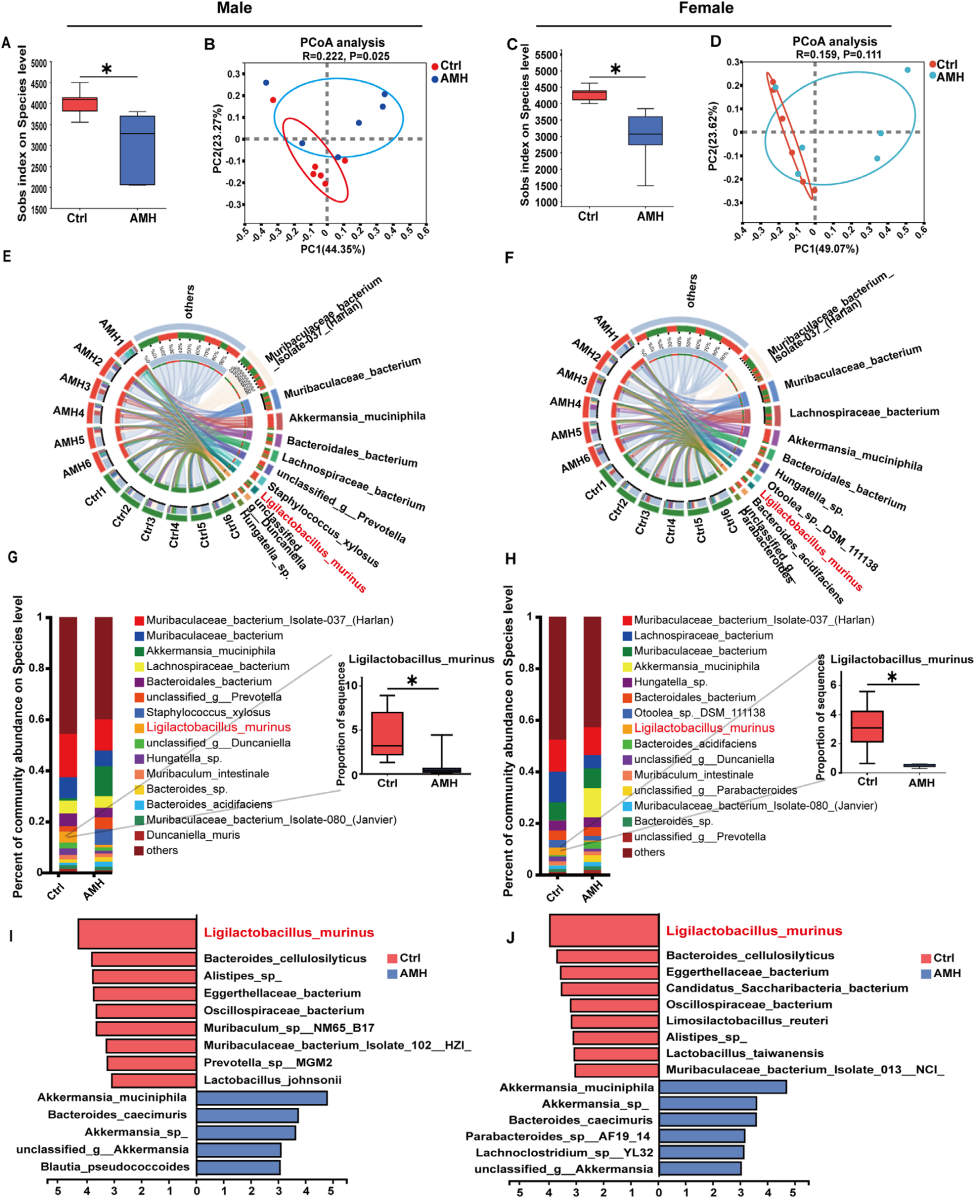
Impacts of perinatal AM exposure on gut microbiota at species level in adult offspring. In animal experiment 1, feces were collected at PNW13 for metagenomic sequencing. A,C) Sobs index shows bacterial community abundance for alpha diversity. B,D) PCoA scatter plots analyze community similarity using UniFrac distances. E,F) Circos plot displays the top 10 abundant bacterial species, with links indicating bacterial strains found in specific samples. G,H) Community bar plot shows the top 15 microbial compositions at the species level, highlighting Ligilactobacillus murinus. I,J) LEfSe analysis uses linear discriminant analysis (LDA) to identify taxa with significant abundance differences between groups, with only taxa having an LDA threshold > 2 shown. Data are expressed as mean ± SEM; significance was assessed with two‐tailed Student's *t*‐test. *n* = 6. ^*^ *p* < 0.05, ^**^ *p* < 0.01.

The gut microbiota composition was assessed using metagenomics. Sobs index showed that species richness was obviously reduced in AM‐exposed male and female offspring (**Figure** **4**A,C). The principal coordinate analysis (PCoA) was performed to evaluate beta‐diversity of gut microbiome signatures and showed significant differences between AM‐exposed offspring and controls by Bray‐Curtis distance (Figure 4B,D). Circos plot was used to display top 10 most abundant bacterial strains. In controls, Lactobacillus dominates noticeably (Green). Lactobacillus abundance was obviously reduced in AM‐exposed offspring (Figure 4E,F). Further analysis showed that gut microbiota at species level had remarkable disruptions in AM‐exposed males and females (Figure 4G,H). Linear discriminant analysis effect size (LEfSe) algorithm was used to analyze bacterial composition and showed that Lactobacillus murinus dominated the communities found in controls while very low abundance in AM‐exposed male and female offspring (Figure 4I,J).

### 2.8 Perinatal AM Exposure Disrupts Gut Microbiota Colonization in Early Life

**Figure 5:**
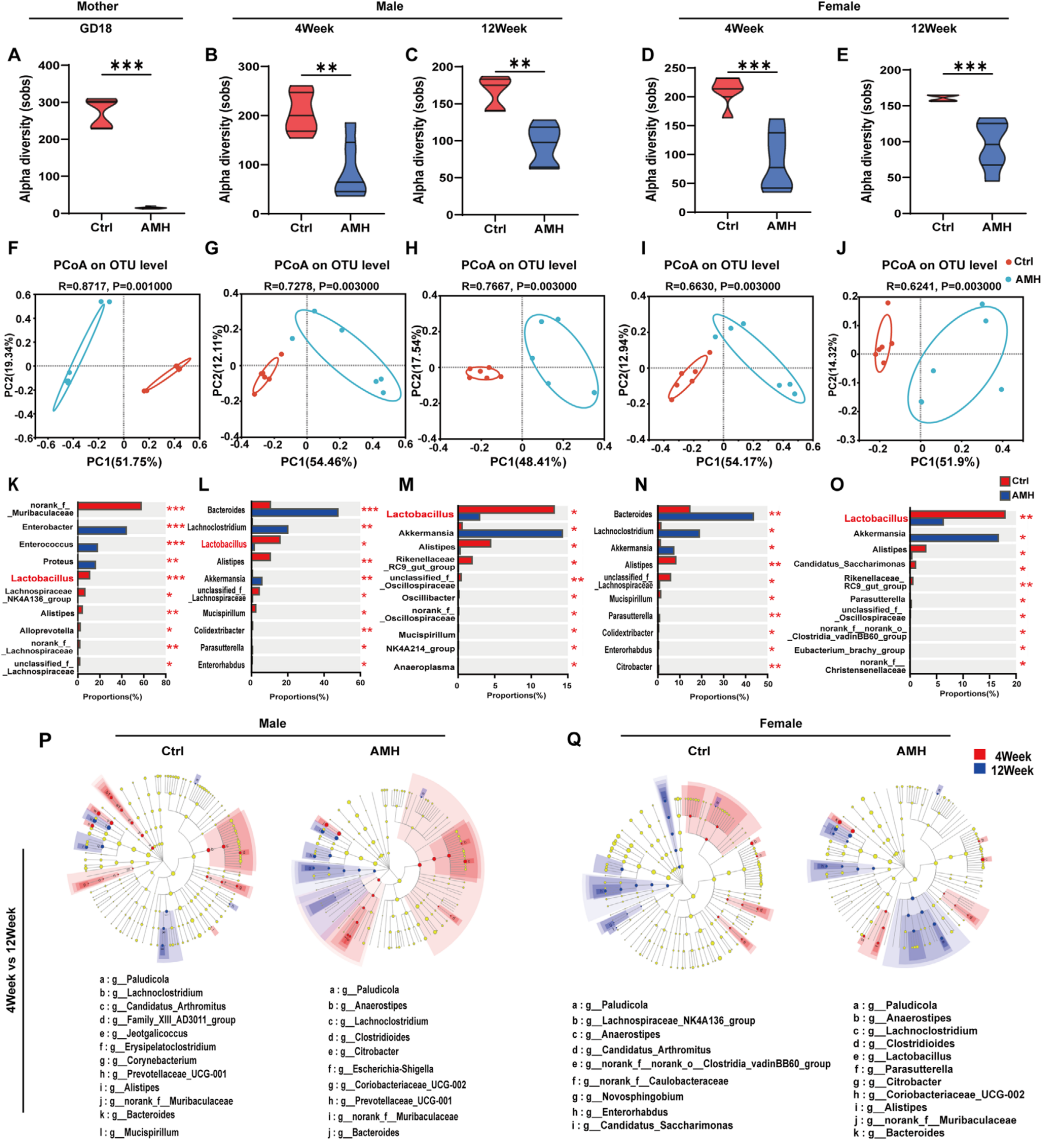
Impacts of perinatal AM exposure on gut microbiota composition in maternal mice, weaned pups, and adult offspring. In animal experiment 1, maternal feces were collected at GD18, and offspring feces at PNW4 and PNW12. Gut microbiota was assessed using 16S rRNA sequencing. A–E) Sobs index indicates bacterial community abundance for alpha diversity. F–J) PCoA scatter plots show bacterial community similarity via UniFrac distances. K–O) Top 10 genera with significant relative abundance differences. P–Q) LEfSe species hierarchical diagram presents species differences across taxonomic levels as a phylogenetic tree. Data are expressed as mean ± SEM; significance was assessed with two‐tailed Student's *t*‐test. *n* = 6. ^*^ *p* < 0.05, ^**^ *p* < 0.01.

To investigate the influence of perinatal AM exposure on gut microbiota colonization, fecal microbiota structure was evaluated at different time points after birth, and gut microbiota function was evaluated in adulthood. As shown in **Figure** **5**A–E, Sobs indices were reduced not only in maternal mice during childbirth but also in weaning pups and adult offspring. Moreover, lower community richness was shown not only in AM‐exposed maternal mice during childbirth but also weaning pups and adult offspring (Figure S5A–E, Supporting Information). The beta‐diversity of gut microbiome signatures was evaluated in maternal mice during childbirth, weaning pups, and adult offspring. A significant difference was shown between AM‐exposed mice and controls (Figure 5F–J). Venn diagrams at the OTU level showed that the number of OTUs was reduced not only in AM‐exposed maternal mice during childbirth but also weaning pups and adult offspring (Figure S5F–J, Supporting Information). Community bar plots were used to visually illustrate the trends of species changes at different time points after birth. At genus level, top 10 most abundant bacterial genera were highlighted. The relative abundance of Lactobacillus was significantly reduced not only in AM‐exposed maternal mice during childbirth but also weaning pups and adult offspring (Figure S5K–O, Supporting Information). Differential testing was employed to assess the variations in microbial composition between AM‐exposed mice and controls. As shown in Figure 5K–O, top 10 bacterial taxa showed significant differences not only in AM‐exposed mice and controls. Further analysis showed that Lactobacillus population was obviously decreased not only in maternal mice during childbirth but also weaning pups and adult offspring (Figure 5K–O). Based on the 16S amplicon sequencing data combined with the eggNOG databases, we predicted the biological functions of the bacteria (Figure S6, Supporting Information). In combination with the eggNOG database, the functions of bacteria were predicted to be mainly involved in metabolism, with lipid transport and metabolism in PNW12 offspring (Figure S6A,B, Supporting Information). Finally, microbial changes were compared between weaning pups and adult offspring. As shown in Figure 5P,Q, the relative abundance of Lactobacillus populations wasn't changed in AM‐exposed mice and controls.

### 2.9 Supplementation with Lactobacillus Murinus Partially Improves Composition of Gut Microbiota in AM‐Exposed Male Offspring

**Figure 6:**
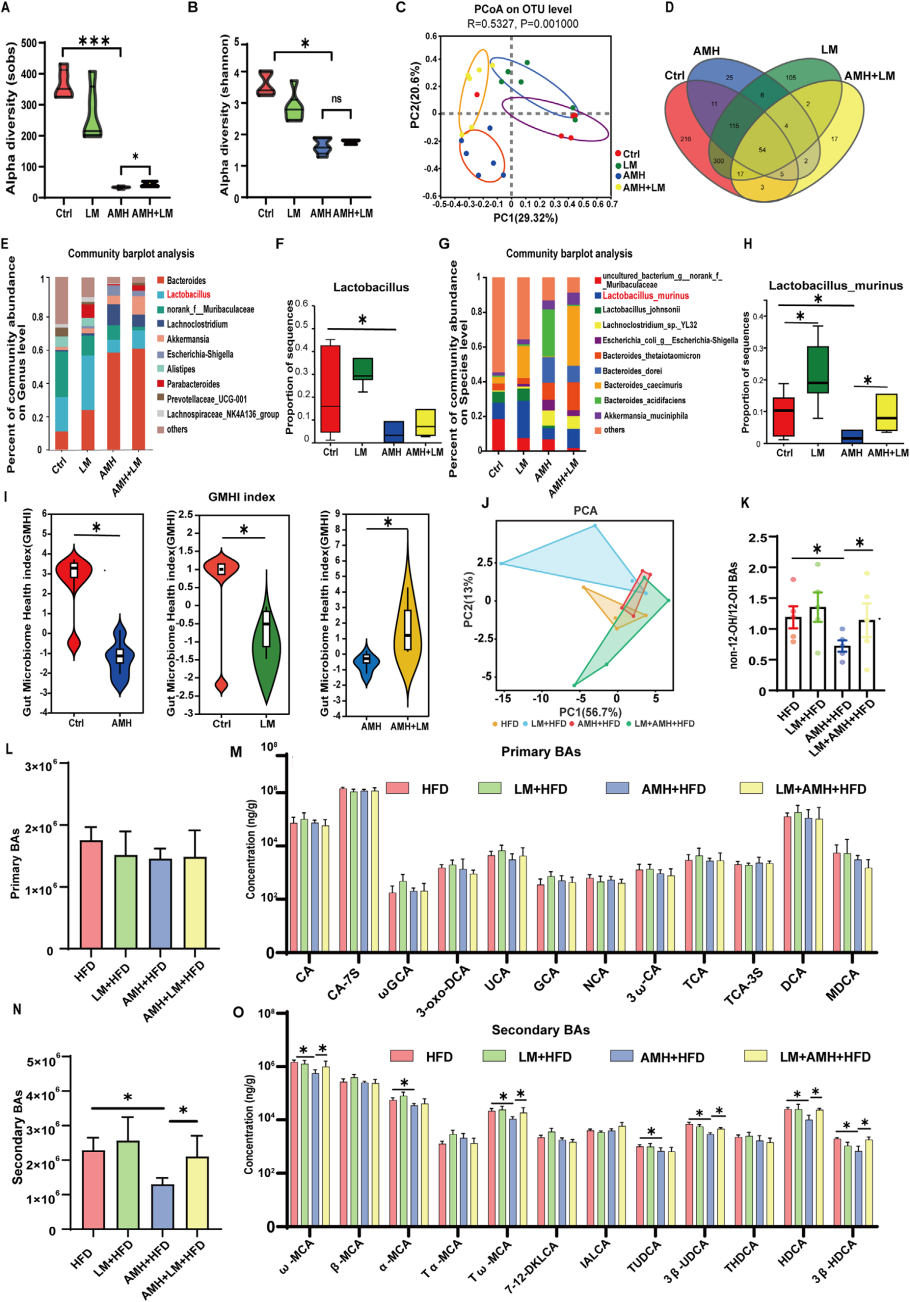
Effects of supplementing Lactobacillus murinus on gut microbiota composition and fecal bile acid metabolism in male offspring. In animal experiment 5, feces were collected at PNW4. A) Sobs index shows bacterial community abundance for alpha diversity. B) Shannon index displays bacterial community diversity. C) PCoA scatterplot depicts community similarity using UniFrac distance. D) Venn diagram shows species count at the OTU level. E) Community bar chart displays top 10 genera. F) Relative abundance of Lactobacillus. G) Community bar chart of top 10 species. H) Relative abundance of Lactobacillus murinus. I) GMHI analyzes group differences. All data are expressed as mean ± SEM; *n* = 6. ^*^ *p* < 0.05, ^**^ *p* < 0.01. At PNW19, cecum contents were collected to test BAs. J) PCA score plots. K) Ratio of non‐12‐OH to 12‐OH BAs. L,N) Primary and secondary BAs. M,O) Primary and secondary BA concentrations. Data are expressed as mean ± SEM; significance was evaluated with two‐sided one‐way ANOVA and post hoc LSD tests. *n* = 4–5. ^*^ *p* < 0.05.

The effect of supplementation with Lactobacillus murinus during lactation on gut microbiota composition was evaluated in AM‐exposed male offspring. Sob and Shannon index showed that supplementing Lactobacillus murinus slightly restored community richness (**Figure** **6**A) without affecting community diversity (Figure 6B). PCoA plot showed the smallest difference between controls and Lactobacillus murinus‐supplemented offspring while there was the largest difference between controls and AM‐exposed offspring. Supplementing Lactobacillus murinus partially reduced the difference between controls and AM‐exposed offspring (Figure 6C). Venn diagram at the OTU level showed that supplementing Lactobacillus murinus did not reverse the number of OTUs (Figure 6D). Community bar plots were used to evaluate the effect of Lactobacillus murinus supplementation on top 10 most abundant species at the genus and species levels in gut microbiota of male offspring (Figure 6E,G). AM‐induced decrease in the proportion of Lactobacillus murinus was partially reversed in Lactobacillus murinus‐supplemented male offspring (Figure 6F,H). The Gut Microbiota Health Index (GMHI) was used to further evaluate gut health and dysbiosis in male offspring. As shown in Figure 6I, supplementing Lactobacillus murinus reversed AM‐induced reduction of GMHI in male offspring.

### 2.10 Supplementation with Lactobacillus Murinus Partially Improves BA Metabolism in AM‐Exposed Male Offspring

The effect of supplementation with Lactobacillus murinus on intestinal BA metabolism was analyzed. Principal component analysis of fecal BA profiles showed a separation among different groups (Figure 6J). Accordingly, the ratio of non‐12‐OH BAs to 12‐OH BAs was reduced in AM‐exposed HFD‐fed male offspring. In addition, supplementing Lactobacillus murinus reversed the decrease in the ratio of non‐12‐OH BAs to 12‐OH BAs in AM‐exposed HFD‐fed male offspring (Figure 6K). Despite no difference on primary BAs, secondary BAs were reduced in AM‐exposed HFD‐fed male offspring (Figure 6L,N). Supplementing Lactobacillus murinus reversed the reduction of secondary BAs in AM‐exposed HFD‐fed male offspring (Figure 6L,N). Next, the effect of supplementation with Lactobacillus murinus on fecal BA composition was analyzed. No difference on primary BAs is shown among different groups (Figure 6M). Secondary BAs, including MCA, UDCA, and HDCA, were decreased in AM‐exposed HFD‐fed male offspring (Figure 6O). Supplementing Lactobacillus murinus reversed the reduction of secondary BAs in AM‐exposed HFD‐fed male offspring (Figure 6O).

### 2.11 Supplementation with Lactobacillus Murinus Attenuates AM‐Evoked Susceptibility to HFD‐Induced Hepatic Lipid Accumulation in Male Offspring

**Figure 7:**
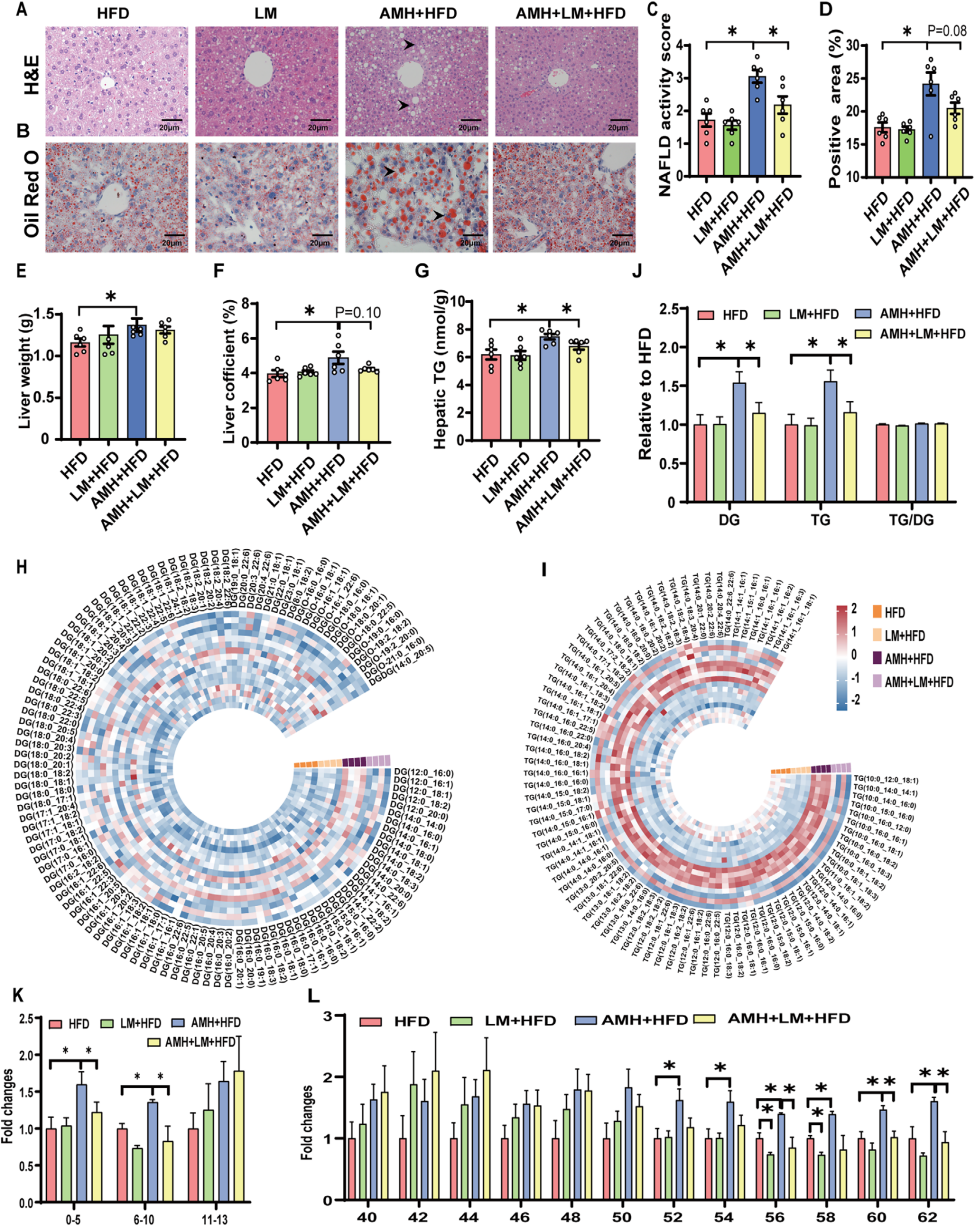
Effects of supplementation with Lactobacillus murinus on HFD‐induced hepatic lipid accumulation in male offspring. In animal experiment 5, hepatic tissues were collected at PNW 19. A) Representative H&E staining photomicrographs at ×400 magnification. B) Hepatic sections stained with Oil Red O at ×400 magnification. C) Quantitative analysis of NAFLD activity score. D) Quantitative analysis of Oil Red O staining. E) Liver weight. F) Liver coefficient. G) Hepatic TG content. H) Ring heat map showing subclass composition of hepatic DGs. I) Ring heat map showing subclass composition of hepatic TGs. J) Hepatic glyceride subclasses. K) Relative abundance of TGs with varying degrees of unsaturation. L) Relative abundance of TGs with different numbers of double bonds. All data are expressed as mean ± SEM; significance was evaluated with two‐sided one‐way ANOVA and post hoc LSD tests. *n* = 4–6. ^*^ *p* < 0.05.

Before HFD feeding, no difference in body weight was shown among different groups (Figure S7A, Supporting Information). After HFD feeding for 6 weeks, body weight was elevated in AM‐exposed HFD‐fed male offspring. Supplementing Lactobacillus murinus attenuated the elevation of body weight in male offspring (Figure S7B, Supporting Information). Food intake was measured, showing no difference among different groups (Figure S7C, Supporting Information). As expected, gWAT was increased in AM‐exposed HFD‐fed male offspring. Supplementing Lactobacillus murinus attenuated the elevation of gWAT in male offspring (Figure S7D, Supporting Information). Despite of no difference in serum TC and LDL, the elevation of serum ALT, AST, and TG was attenuated in Lactobacillus murinus‐administered male offspring (Figure S7E–H,J, Supporting Information). The reduction of serum HDL‐C content was alleviated in Lactobacillus murinus‐administered male offspring (Figure S7I, Supporting Information). NAFLD activity scores were lower in L. murinus–supplemented AMH‐exposed HFD‐fed mice than those in AM‐exposed HFD‐fed controls (**Figure** **7**A,C). Fewer hepatic lipid droplets, determined by Oil Red O staining, were shown in Lactobacillus murinus‐supplemented AMH‐exposed HFD‐fed male offspring than those in AMH‐exposed HFD‐fed controls (Figure 7B,D). Despite no effect on liver weight (Figure 7E), supplementing Lactobacillus murinus attenuated the elevation of liver coefficient in male offspring (Figure 7F). Moreover, supplementing Lactobacillus murinus reversed AM‐exposed HFD‐induced elevation of hepatic TG content in male offspring (Figure 7G). Next, the effect of supplementing Lactobacillus murinus on hepatic lipid profiles was analyzed. The volcano plots and OPLS‐DA score maps showed the differential lipid metabolites between HFD‐fed and AMH‐exposed HFD‐fed male offspring (Figure S7K,N, Supporting Information), between Lactobacillus murinus‐supplemented AMH‐exposed HFD‐fed and AM‐exposed HFD‐fed male offspring (Figure S7L,O, Supporting Information), and between Lactobacillus murinus‐supplemented HFD‐fed and HFD‐fed male offspring (Figure S7M,P, Supporting Information). The OPLS‐DA score plot showed differences among different groups (Figure S7Q, Supporting Information). Qualitative analysis showed that supplementing Lactobacillus murinus attenuated AM‐exposed HFD‐induced elevation of hepatic total lipids and glycerolipids in male offspring (Figure S7R, Supporting Information). Finally, the effect of supplementing Lactobacillus murinus on hepatic glycerolipids was analyzed. Circular heat maps further showed that AM‐exposed HFD‐induced elevation of hepatic DG and TG contents was reversed in Lactobacillus murinus‐administered male offspring (Figure 7H,I). As expected, hepatic DG and TG contents were elevated in AM‐exposed HFD‐fed male offspring. Supplementing Lactobacillus murinus reversed AM‐exposed HFD‐induced elevation of hepatic DG and TG in male offspring (Figure 7J). Further analysis for structural diversity of TGs revealed that TGs with a low number of carbons (0–10) (Figure 7K) and a high number of double bonds (>50) (Figure 7L) predominated in AM‐exposed HFD‐fed male offspring. Supplementing Lactobacillus murinus attenuated AM/HFD‐induced elevation of hepatic TG contents (Figure 7K,L).

## 3 Discussion

This study investigated the influence of perinatal AM exposure on susceptibility to HFD‐induced hepatic lipid accumulation in adulthood. The major innovative findings are as follows: first, perinatal AM exposure exacerbates HFD‐evoked hepatic lipid deposition in adulthood; second, perinatal AM exposure promotes dietary lipid absorption by disrupting intestinal BA metabolism; third, perinatal AM exposure interferes with early‐life gut microbiota colonization and persistently disrupts adulthood gut microbiota structure and function; finally, supplementing Lactobacillus during lactation improves gut microbiota colonization and intestinal BA metabolism, and attenuates HFD‐induced hepatic lipid deposition. These findings provide evidence that early‐life gut microbiota colonization influences the susceptibility to hepatic lipid accumulation in adulthood.

Hepatic lipid accumulation is one of the critical features of MAFLD.^[^ ^33^ ^]^ Increasing data have demonstrated that gut microbiota is considered a key regulatory factor for hepatic lipid deposition and metabolism.^[^ ^34^ ^]^ In this study, we evaluated the influence of perinatal AM exposure on gut microbiota colonization and susceptibility to adulthood hepatic lipid accumulation in HFD‐fed mice. The results showed that HFD‐induced hepatic lipid deposition was exacerbated in AM‐exposed adult males but not females. Hepatic lipid profile was analyzed and revealed that HFD‐evoked elevation of total lipids, fatty acyls, and glycerol lipids was aggravated in AM‐exposed adult males but not females. Accumulating data have demonstrated that early‐life exposure to adverse environment elevates susceptibility to adulthood MAFLD by disrupting epigenetic reprogramming in the developing liver.^[^ ^10^, ^35^, ^36^ ^]^ In the current study, the impact of perinatal AM exposure on hepatic lipid metabolic genes was analyzed. Transcriptome results showed that no lipid metabolism‐related pathway was enriched in AM‐exposed HFD‐fed offspring. RT‐PCR verified that hepatic lipid metabolic genes, including hepatic lipid synthesis, β‐oxidation, lipid degradation, and lipid transport, were not altered in AM‐exposed HFD‐fed adult offspring. These results indicate that perinatal AM exposure does not influence hepatic lipid metabolism. Next, we performed a subacute experiment to test the influence of AM exposure on intestinal lipid absorption. Postprandial TG response experiment showed that dietary lipid absorption was elevated in AM‐administered males but not AM‐administered females. These results suggest that perinatal AM exposure exacerbates HFD‐fed hepatic lipid deposition by increasing dietary lipid absorption.

It is widely accepted that dietary lipid absorption is modulated by intestinal BAs.^[^ ^37^, ^38^, ^39^ ^]^ Primary BAs, such as CA and CDCA, are beneficial for intestinal lipid absorption.^[^ ^40^ ^]^ By contrast, secondary BAs, such as HDCA and MCA, facilitate elimination of dietary lipids from small intestine.^[^ ^41^ ^]^ In this study, the influences of perinatal AM exposure on the composition of primary and secondary BAs were analyzed. Although primary BAs contents were not altered, secondary BAs, including ω‐MCA, Tα‐MCA, Tω‐MCA, 6‐ketoLCA, IALCA, TUDCA, 3β‐UDCA, THDCA, and 3β‐HDCA, were reduced in AM‐exposed HFD‐fed adult males but not in females. Numerous studies demonstrate that gut microbiota promotes metabolism of primary BAs to produce secondary BAs.^[^ ^42^, ^43^ ^]^ The present study evaluated the influences of perinatal AM exposure on the composition of gut microbiota using metagenomics. Our results showed that species richness and diversity were reduced in AM‐exposed adult offspring. Detailed analysis showed that Lactobacillus murinus was obviously decreased in AM‐exposed adult offspring. It is known that Lactobacillus expresses bile salt hydrolase (BSH), which deconjugates bile acids (e.g., TCA, GCA) into free bile acids (e.g., CA, CDCA). These free bile acids are further metabolized by gut microbiota (Clostridium) into secondary bile acids (e.g., DCA, LCA).^[^ ^44^, ^45^ ^]^ Thus, the decreased Lactobacillus reduces BSH activity, elevating conjugated bile acids that enhance lipid digestion and absorption due to their superior emulsifying properties. These results suggest that perinatal AM exposure inhibits primary BA metabolism to reduce secondary BA production probably through altering the structure and function of adulthood gut microbiota.

It is well known that gut microbiota is colonized in early life.^[^ ^25^, ^28^ ^]^ Increasing data have demonstrated that maternal microbiome during childbirth determines composition of gut microbiota in adult offspring.^[^ ^46^, ^47^ ^]^ To investigate the impacts of perinatal AM exposure on colonization of gut microbiota, the composition of gut microbiota was analyzed in maternal dams during childbirth and offspring at different stages after birth. Our results displayed that richness and diversity of microbial community were obviously reduced not only in AM‐exposed maternal dams during childbirth but also weaning pups and adult offspring. Moreover, Lactobacillus population was decreased not only in AM‐exposed maternal dams during childbirth but also weaning pups and adult offspring. Finally, microbial changes were compared between weaning pups and adult offspring, and showed that the relative abundance of Lactobacillus population was not changed. These results suggest that early life is a critical period for gut microbiota colonization, and perinatal AM exposure may permanently alter the structure and function of adult gut microbiota by disrupting gut microbiota colonization in early life.

In this study, our results found that perinatal AM exposure altered gut microbiota colonization in male and female offspring. However, changes in intestinal bile acid metabolism occurred only in male offspring but not in female offspring. These results indicate that alteration of gut microbiota promotes dietary lipid absorption in a gender‐dependent manner. Several reports from other laboratory showed a similar gender difference in the susceptibility to diet‐evoked fatty liver disease.^[^ ^48^, ^49^, ^50^ ^]^ The mechanism underlying gender differences may be related to sex hormones.^[^ ^51^ ^]^ On the other hand, several early studies have demonstrated that there are gender differences in bile acid metabolism.^[^ ^52^, ^53^ ^]^ The exact mechanisms of gender differences in dietary lipid absorption remain unclear and need be further explored in future research.

The results of this study have significant translational value in clinical practice. Indeed, Lactobacillus, a dominant gut microbiota that encodes bile salt hydrolase, converts primary BAs to secondary BAs.^[^ ^54^, ^55^ ^]^ The present study showed that Lactobacillus was reduced not only in AM‐exposed weaning pups but also adult offspring. We used metagenomic screening to identify the most contributory strain, Lactobacillus murinus, and supplemented with this strain from PND8 to PND28 to directly observe its effects on gut microbiota colonization and dietary lipid absorption. The results indicated that supplementation with Lactobacillus murinus improved not only early‐life gut microbiota colonization but also adulthood bile acid metabolism and the susceptibility to hepatic lipid deposition. An earlier study found that maternal supplementation with Lactobacillus during lactation diminished respiratory syncytial virus‐induced goblet cell hypertrophy and mucus production in offspring.^[^ ^56^ ^]^ A recent report showed that gestational supplementing Lactobacillus prevented offspring from dextran sulfate sodium‐induced colitis.^[^ ^57^ ^]^ These results suggest that supplementing Lactobacillus during lactation might be a potential method to improve early‐life gut microbiota colonization and adulthood HFD‐induced hepatic lipid deposition.

There were several limitations in our work. First, this study found that disruptions of early‐life gut microbiota colonization elevated susceptibility to adulthood HFD‐induced hepatic lipid accumulation. Next work is required to further evaluate the influence of early‐life gut microbiota colonization on susceptibility to adulthood high‐fructose‐induced MAFLD. Second, it has been accepted that MAFLD is accompanied by overweight or obesity, insulin resistance, and metabolic dysregulation. Additional work should explore the impact of early‐life gut microbiota colonization on susceptibility to adulthood obesity and insulin resistance. Third, this study found that perinatal AM exposure equally interfered with early‐life gut microbiota colonization in male and female offspring. Unexpectedly, intestinal bile acid metabolism is disrupted only in males but not females. Moreover, the increased susceptibility to HFD‐induced hepatic lipid deposition occurred only in AM‐exposed males but not females. The differences in gender susceptibility need to be further explored in future research. Fourth, 16S rRNA sequencing‐based functional prediction of the microbiota revealed alterations in lipid transport and metabolism functions. Bile acids are one of the critical factors involved in intestinal lipid transport and metabolism. In addition, gut barrier function and inflammation are also important mechanisms in the pathogenesis of MAFLD. We plan to include a more comprehensive analysis of gut barrier function and inflammation in future studies to further elucidate the mechanisms underlying MAFLD development. Future research could explore the effects of other probiotics, prebiotics, or fecal microbiota transplantation on gut microbiota and hepatic lipid accumulation.

## 4 Conclusion 

In summary, our results indicate that perinatal AM exposure disrupts early‐life colonization of gut microbiota, leading to a permanent reduction in gut microbial diversity and the impairment of microbial community structure and function. We demonstrate that disruptions of early‐life gut microbiota colonization elevate the susceptibility to adulthood hepatic lipid accumulation. Mechanistically, disruption of gut microbiota colonization inhibits primary BA metabolism and reduces secondary BA production, thereby promoting dietary lipid absorption. Supplementing Lactobacillus during lactation alleviates early‐life gut microbiota colonization and adulthood hepatic lipid deposition. Our results demonstrate that early‐life gut microbiota colonization influences susceptibility to adulthood hepatic lipid accumulation through disrupting intestinal BA metabolism. These results provide new insights into the mechanisms underlying the developmental origin of adulthood MAFLD. Supplementing Lactobacillus during lactation may be an efficient strategy for the prevention of adulthood MAFLD.

## 5 Experimental Section

### Animal Experiments

**Experiment 1**. Eight‐week‐old C57BL/6J mice (females: 18–20 g; males: 22–24 g) were obtained from Beijing Vital River Laboratory Animal Technology Co., Ltd. Mice underwent a 2‐week acclimation period before the breeding protocols commenced. They were housed in a Specific Pathogen Free (SPF) environment under a 12‐h light/dark cycle at 22 ± 3 °C with 50 ± 5% humidity. Food and water were provided ad libitum. For breeding, females and males were paired overnight at a 4:2 ratio from 8 p.m. to 7 a.m., and mating was confirmed by the presence of vaginal plugs, marking gestational day (GD)0. Maternal antibiotic exposure was primarily used to prevent infections and treat complications, typically occurring around the time of birth. In our mouse model, AM was administered to maternal dams from GD13 through postnatal day (PND)7 to closely mimic the clinical timing of exposure.^[^ ^58^ ^]^ On GD13, maternal mice were divided into three groups: control (Ctrl), low AM (AML, 20 mg kg^−1^ d^−1^), and high AM (AMH, 200 mg kg^−1^ d^−1^). Control mice received sterile water by gavage, while AML and AMH groups received corresponding doses of AM from GD13 to PND7. AM was dissolved in sterile water. Human dosages range from 500 to 1500 mg d^−1^ (https://www.drugs.com/dosage/amoxicillin.html). Based on body surface area calculations,^[^ ^59^ ^]^ mouse dosages were ≈50–150 mg kg^−1^ d^−1^. Pups weaned on PND28 and were fed normal chow (3.44 kcal g^−1^: 24% protein, 13% fat, 63% carbohydrates). At postnatal week (PNW)13, pups were randomly split into normal chow (NC) and high‐fat diet (HFD) subgroups. The HFD included 14% protein, 42% fat, 44% carbohydrates, and 0.2% cholesterol (TP 26304, TrophicDiet, Nantong, China) for 6 weeks. Offspring were weighed regularly, and fecal samples collected throughout the study.

**Experiment 2**. Twelve‐week‐old male and female C57BL/6J mice were randomly divided into two groups. In the control group, mice were administered with equal capacity sterile water by gavage. In the AMH group, mice were administered with AM (AMH, 200 mg kg^−1^ d^−1^) by gavage for 6 days. After fasting for 4 h, baseline blood samples were collected from the mice via tail bleeding. Next, the mice were administered by gavage with olive oil at a dose of 10 µL g^−1^ body weight. Blood was drawn by tail bleeding at 1, 2, 4, and 6 h post‐oil bolus. To ensure sufficient yield for downstream analysis, 30–40 µL of whole blood was collected, and serum was isolated by centrifugation at 3500 rpm for 15 min at 4 °C, yielding ≈10 µL serum. Mice had access to water but not food for the entire experiment.

**Experiment 3**. The same AM exposure as in animal experiment 2 was given. After six days of AM exposure, animals were fasted overnight, refed 60% HFD for 2 h, and then euthanized. Intestinal tissue was collected and stored in paraformaldehyde for further analysis.

**Experiment 4**. The same AM exposure as in animal experiment 2 was given. The experimental process was performed as described by the literature.^[^ ^60^ ^]^ Briefly, after six days of AM exposure, mice were fasted for 4 h and administered with BODIPY 500/510 C1, C12 FAs (2 µg g^−1^ body weight), and olive oil (10 µL g^−1^ body weight) for 2 h, and then euthanized. The resected small intestine was embedded and frozen at cutting temperature (OCT) for further analysis.

**Experiment 5**. The procurement and housing conditions of the animals were consistent with those in Experiment 1. On GD13, maternal mice were randomly separated into two groups: control (Ctrl) or high AM (AMH, 200 mg kg^−1^ d^−1^) group. Maternal mice were administered by gavage with equal capacity sterile water or AM (200 mg kg^−1^ d^−1^) from GD13 to PND7. On PND8, male offspring in the control group were randomly divided into two subgroups: half remained as the Ctrl group and the other half was administered with Lactobacillus murinus by gavage and designated as the LM group. Similarly, male offspring in the AMH group were randomly divided into two subgroups: half remained as the AMH group, and the other half was administered Lactobacillus murinus by gavage and designated as the AMH+LM group. On PNW13, all male offspring were fed with HFD for 6 weeks. All offspring were regularly weighed and fecal samples were collected throughout study.

### Metagenomics Sequencing

This experiment was conducted as described by literature.^[^ ^61^ ^]^ Briefly, each sample was prepared with 700 ng DNA. PCR amplification and purification were carried out using the AMPure XP system. DNA concentration was measured with the Qubit DNA Assay Kit on a Qubit 2.0 Fluorometer (Life Technologies, CA, USA) and diluted to 2 ng mL^−1^. The insert size of library was evaluated using the Agilent Bioanalyzer 2100 system. Accurate concentration (>3 nm) of the library was verified by qPCR. Post cluster generation, the library preparations underwent sequencing on an Illumina HiSeq 4000 platform, resulting in 150‐bp paired‐end reads. Diversity Analysis: alpha diversity (Shannon and Sobs indices) and beta diversity (Bray‐Curtis dissimilarity) were computed using QIIME2. Principal Coordinate Analysis (PCoA) was performed to visualize sample clustering. Differential Abundance: differential abundance of species between groups was assessed using DESeq2, with significance thresholds set at *p* < 0.05 and fold change ≥ 2. The testing was assisted by the Shanghai Majorbio Bio‐Pharm Technology Co., Ltd. (Shanghai, China).

### Hepatic Lipidomic Analysis

Hepatic tissues were thawed on ice, mixed for ≈10 s, and then processed using a centrifuge at 3000 rpm at 4 °C for 5 min. Each 20 mg sample was homogenized with a 1 mL mixture containing methanol, MTBE and an internal standard mixture, and then mixed for 15 min. To the mixture, 200 µL water was added and stirred for 1 min before being centrifuged at 12 000 rpm at 4 °C for 10 mins. The supernatant, amounting to 500 µL, was extracted and concentrated after centrifugation. The concentrated powder was dissolved in 200 µL solvent B (acetonitrile: isopropanol at a 1:9 vol ratio using 10 mm ammonium formate and 0.1% formic acid) and used for LC‐MS/MS analysis. Multivariate Analysis: Principal Component Analysis (PCA) and Partial Least Squares‐Discriminant Analysis (PLS‐DA) were performed using MetaboAnalyst 5.0 to identify lipid species contributing to group separation. Univariate Analysis: Student's *t*‐test or ANOVA was used to identify significantly altered lipids (*p* < 0.05, fold change ≥ 2). The testing was facilitated by Wuhan Metware Biotechnology Co., Ltd. (Wuhan, China).

### Quantitative Analysis of Bile Acids

Cecal contents (20 mg) were mixed with 10 µL of internal standard mixture solution (1 µg mL^−1^) and 200 µL of methanol/acetonitrile (v/v = 2:8) and then homogenized. The sample was vortexed at 2500 rpm for 10 mins and stored at −20 °C for 10 mins to facilitate protein precipitation. Finally, the mixture was centrifuged at 12 000 rpm for 10 mins at 4 °C. The supernatant was transferred to a new plastic microtube and concentrated using a concentrator (CentriVap, LABCONCO, USA). After concentration, the samples were reconstituted in 100 µL of a 50% methanol‐water solution for subsequent LC‐MS/MS analysis with an Applied Biosystems 6500 triple quadrupole (QTRAP 6500+, SCIEX, USA). The HPLC column used was a Waters ACQUITY UPLC HSS T3 C18 (100 mm × 2.1 mm, 1.8 µm). Quantification: BAs were quantified using a standard curve and normalized to internal standards. Testing was assisted by Wuhan MetWare Biotechnology Co., Ltd. (Wuhan, China).

### Statistical Analysis

Statistical analyses were conducted using SPSS 23.0, with data reported as mean ± SEM. A two‐tailed Student's *t*‐test compared two groups, while one‐way ANOVA was used for multiple groups. When significant differences were found, post hoc LSD tests compared the control and AM exposure groups. The Kruskal‐Wallis H test analyzed non‐normally distributed data. Mouse body weight was assessed using two‐way ANOVA. GraphPad Prism 8.0 was used for graphical representations and visualizations. A *p*‐value of less than 0.05 indicated statistical significance.

Additional information is described in the Supporting Information.

## Conflict of Interest

The authors declare no conflict of interest.

## Ethics Approval and Consent to Participate

The animal protocol was approved by the Animal Care and Use Committee of Anhui Medical University (Ethical approval no. LLSC20200973). All animal experiments adhered strictly to animal ethical standards.

## Author Contributions

Y.‐Y.Z. and X.D. contributed equally to this work. D.X.X. and H.W. conceived and designed the experiments. Y.Y.Z., X.D., H.Z., and Z.Y.L. performed the majority of animal experiments. Y.Y.Z. and X.D. completed the pathological experiments. Y.Y.Z., B.W., Y.P.S., and Y.H.Z. completed the data analysis for 16S rRNA sequencing, metagenomic sequencing, and RNA sequencing. Y.Y.Z., X.L., Z.B.L., and Y.C.H. completed the data analysis for targeted metabolomics. D.X.X. and Y.Y.Z. drafted the original manuscript. All authors contributed to and have approved the final manuscript.

## Supporting information



Supporting Information

Supporting Information

## Data Availability

The data that support the findings of this study are available from the corresponding author upon reasonable request.
