# Supplementary material for: Gut Microbiota Colonization in Early Life Influences Susceptibility to Adulthood Hepatic Lipid Accumulation in High‐Fat‐Diet‐Fed Mice
Source: Adv Sci (Weinh). 2025 Apr 7;12(21):2412827. doi: 10.1002/advs.202412827 (PMC12140298; doi:10.1002/advs.202412827)
Supplement: Supplementary file 1 — Supporting Information [file ADVS-12-2412827-s002.docx]

Gut Microbiota Colonization in Early Life Influences Susceptibility to Adulthood Hepatic Lipid Accumulation in High-Fat-Diet-Fed Mice

Yan-Yan Zhu,^1,2, #^ Xin Dong,^1,2, #^ Hao Zhou,^1,2^ Ze-Yan Li,^1,2^ Bo Wang,^1,2^ Ya-Ping Song,^1,2^ Zhi-Bing Liu,^1,2,3^ Xue Lu,^1,2^ Yi-Hao Zhang,^1,2^ Yichao Huang, ^1,2^ Hua Wang,^1,2, *^ De-Xiang Xu^1,2, *^

**Supplementary methods………………………………………………………………1**

**Supplementary figures…………………………………………………………………7**

**Supplementary references……………………………………………………………21**

**Supplementary methods**

**Reagents and Chemicals**

Amoxicillin (sodium salt, HY-B0467, CAS No. 34642-77-8; ≥98.23%) was sourced from Med Chem Express (USA). Biochemical assay kits for alanine aminotransferase (ALT), aspartate aminotransferase (AST), triglyceride (TG), total cholesterol (TC), high-density lipoprotein cholesterol (HDL-C) and low-density lipoprotein cholesterol (LDL-C) were from Zhejiang Yilikang Biotechnology Co., Ltd. (Zhejiang, China). Triglyceride assay kit (BC0625) was obtained from Beijing Solarbio Science & Technology Co., Ltd. (Beijing, China). TRIzol (Cat. 15596026) reagents were procured from Thermo Fisher Scientific Inc. (USA). Reverse transcriptase (Cat. 10109118001) and real-time PCR (Cat. 04887352001) reagents were sourced from Roche Diagnostics GmbH (Switzerland). Chemicals for lipidomic analysis, including methanol (Cat. 106007), acetonitrile (Cat. 113358), formic acid (98%, Cat. 00940), and isopropanol (Cat. 101040) were from Merck (Germany). Ammonium formate (Cat. A11550) and dichloromethane (Cat. AC6100500) were from Fisher Chemicals (USA). Standard substance for 12:0 Lyso PC (Cat. 855475), Cer (d18:1/4:0) (Cat. 860524), PC (13:0/13:0) (Cat. 850340), DG (12:0/12:0) (Cat. 800812) and TG (17:0/17:0/17:0) (Cat. 860903) were from Avanti Polar Lipids (USA).

**Bacterial strains and growth conditions**

This experimental process referred to the literature method ^[1]^. In summary, Lactobacillus murinus lyophilized powder (Shanghai Preservation Biology Center, Shanghai, China) was reconstituted in 0.5 mL of Man Rogosa Sharpe (MRS) medium (Shanghai Ruichu Biotechnology Co., Ltd, Shanghai, China). The resulting bacterial suspension was plated on blood agar, where colonies developed within approximately 24 hours. Individual colonies were then transferred to MRS medium and incubated at 37°C under anaerobic conditions. After 12-16 hours of growth, the cultures were assessed for optical density at 600 nm (OD600), which ranged from 0.64 to 0.8, corresponding to a bacterial concentration of 1×10^9 CFU/mL as determined by plate counting. Following this, the cultures were centrifuged, washed, and resuspended in saline. The experimental group was administered live bacteria at a dose of 1×10^8 organisms per mouse per day, while the control group received an equivalent volume of saline. L. murinus stocks, preserved in MRS medium containing 25% glycerol, were stored at -80°C for future use.

**Indirect calorimetry**

On PNW 17, 5-6 mice per group were placed in individual metabolic cages (TSE Systems, Germany) to monitor various metabolic parameters. Each mouse was allowed to acclimate in its chamber for 48 h. After the acclimation period, various metabolic parameters were measured at 25°C and under a 12-hour light/dark cycle. Throughout the experiment, all mice had continuous access to both food and water. Physical activity, liquid consumption, food intake, body weight, and respiratory functions (O_2_ consumption and CO_2_ production) were recorded every 2 mins following the initial acclimation. The respiratory exchange ratio (RER) was calculated using the formula VCO_2_/VO_2_. Energy expenditure (EE) (Kcal/h) = (3.815 + 1.232 × RER) × VO_2_ (ml/min). Food consumption was specifically measured within the metabolic chambers. All collected data were averaged and presented for both dark and light phases over the 2 consecutive days of monitoring.

**Tissue and feces sample collection**

At specified intervals, the mice were anesthetized, and tissues were meticulously removed. The collected tissue samples were weighed and divided into two groups: Some are immobilized in 4% paraformaldehyde for subsequent histological examination, while others are fast-seeded plasma fecal particles and intestinal contents in liquid nitrogen, flash-frozen in liquid nitrogen, and stored at -80°C until analysis is required.

**Measurements of biochemical parameters**

For the biochemical parameters, including plasma ALT, AST TG, TC, HDL-C and LDL-C, blood samples were centrifuged to separate plasma. Biochemical parameters were measured using an automatic biochemical analyzer (Dirui CS-T300, Changchun, China).

**Hepatic TG measurement**

Hepatic tissue was homogenized in a 1:1 mixture of N-heptane and isopropanol. Hepatic homogenates were then centrifuged at 4°C at 8000g for 10 min. The supernatant was separated, and TG content was detected with commercial TG kit (Beijing Solarbio Science & Technology Co., Ltd.).

**Histological examination and Oil Red O staining**

Mouse hepatic tissue blocks were immersed in 4% paraformaldehyde for 24 hours, followed by dehydration and embedding in paraffin. The sections were stained sequentially with hematoxylin and eosin. Two researchers blindly scored the tissues, evaluating ballooning (score 0-2), steatosis (score 0-3), and lobular inflammation (score 0-3) ^[2]^. Oil Red O stain with 10 μm hepatic tissue sections, and tissue photographs were scanned using a pathology slide scanner.

**Real-time RT-PCR**

Total RNAs were extracted from hepatic tissue using TRIzol reagent. Total RNAs were treated with RNase-free DNase and then reverse transcribed with reverse transcriptase. Real-time RT-PCR was performed with a LightCycler 480 SYBR Green qPCR master mix. All primers were presented in key resources table.

**High-throughput RNA sequencing**

Total RNA was isolated from matched samples with the TRIzol reagent. RNA-seq libraries were generated using the VAHTS mRNA-seq V3 Library Prep Kit. Quality control procedures included the use of BioAnalyzer and Qubit. Sequencing of all libraries was executed on the Illumina NovaSeq 6000 system. Post quality control, sequence alignment was carried out using STAR. The raw read counts for annotated genes were normalized and subjected to differential expression analysis with DEseq2 v1.40.2, applying thresholds of p <0.05 and fold change≥2. We use Gene Ontology (GO) and Kyoto Encyclopedia of Genes and Genomes (KEGG) pathway. Eenrichment analyses were performed using the clusterProfiler package in R. The tests and analyses were assisted by the Shanghai Personal Biotechnology Cp. Ltd.

**Postprandial triglyceride response assay**

Postprandial triglyceride response assay was performed as described by literature ^[3]^. In the animal experiment 2, serum TG was measured with the kits described above.

**16S rRNA sequencing**

Microbial genomic DNA was extracted from fecal samples using the QIAamp Fast DNA Stool Mini Kit (Qiagen Ltd., Hilden, Germany). The V3–V4 region of the 16S rRNA gene was amplified with the universal primers 341F and 806R. Using QIIME 2 (version 2020.2) to analyze the raw sequences. Initial reads were qualitatively filtered, de-noised and assembled. The optimized-HiFi reads were clustered into operational taxonomic units (OTUs) using UPARSE 7.1 with 97% sequence similarity level. The most abundant sequence for each OTU was selected as a representative sequence. The functional potential of the intestinal microbiota was predicted by PICRUSt2. The testing was assisted by the Shanghai Majorbio Bio-Pharm Technology Co., Ltd. (Shanghai, China). All data were demonstrated on the Majorbio I-Sanger Cloud Platform (<https://cloud.majorbio.com/>).

**Supplementary figures**


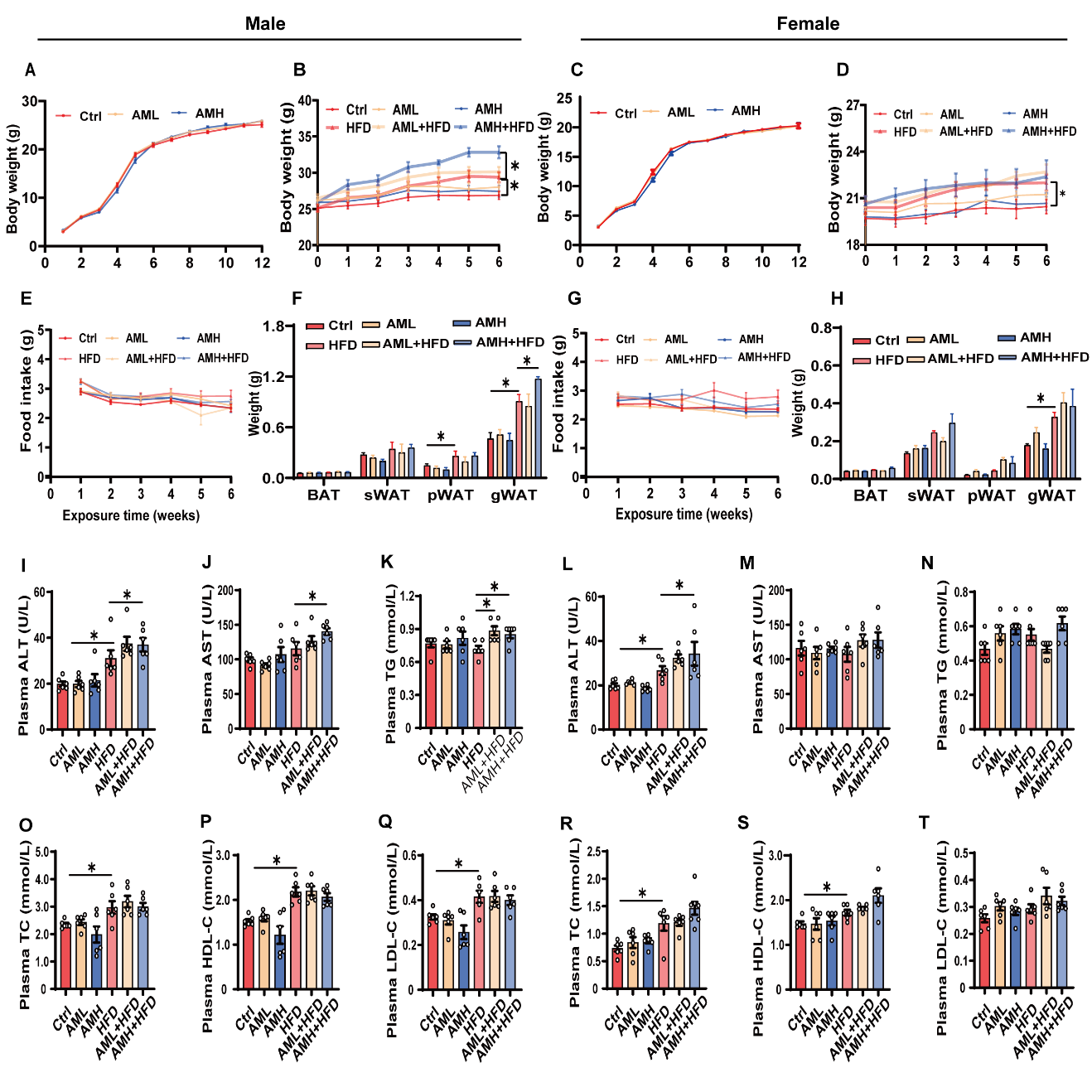


**Figure S1. Impact of perinatal AM exposure on fat weight and plasma lipids in HFD-fed adult offspring**

Maternal mice were administered with either NS, or low dose AM (AML, 20 mg/kg) or high dose AM (AMH, 200 mg/kg) by gavage from GD13 to PND7. All offspring were fed with normal chow (NC) from PND28 to PNW12. At PNW13, half were maintained on normal chow and half were fed with HFD for 6 weeks. At PND19, fat tissues and blood plasma were collected. (A and C) Body weight changes before HFD feeding. (B and D) Body weight changes after HFD feeding. (E and G) Food intake after HFD feeding was compared among different groups. (F and H) Fat weight at PNW19 was compared among different groups. (I, J, L and M) Plasma ALT and AST were measured. (K and N) Plasma TG was analyzed. (O-T) Plasma TC, HDL-C and LDL-C were measured. Abbreviations: ALT, alanine aminotransferase; AST, aspartate aminotransferase; HDL-C, high density lipoprotein cholesterol; LDL-C, low density lipoprotein cholesterol; TC, total cholesterol; TG, triglyceride; Data are presented as mean ± SEM, statistical significance was evaluated by two-sided one-way ANOVA with post hoc LSD tests. n = 5-6. *p < 0.05.

**
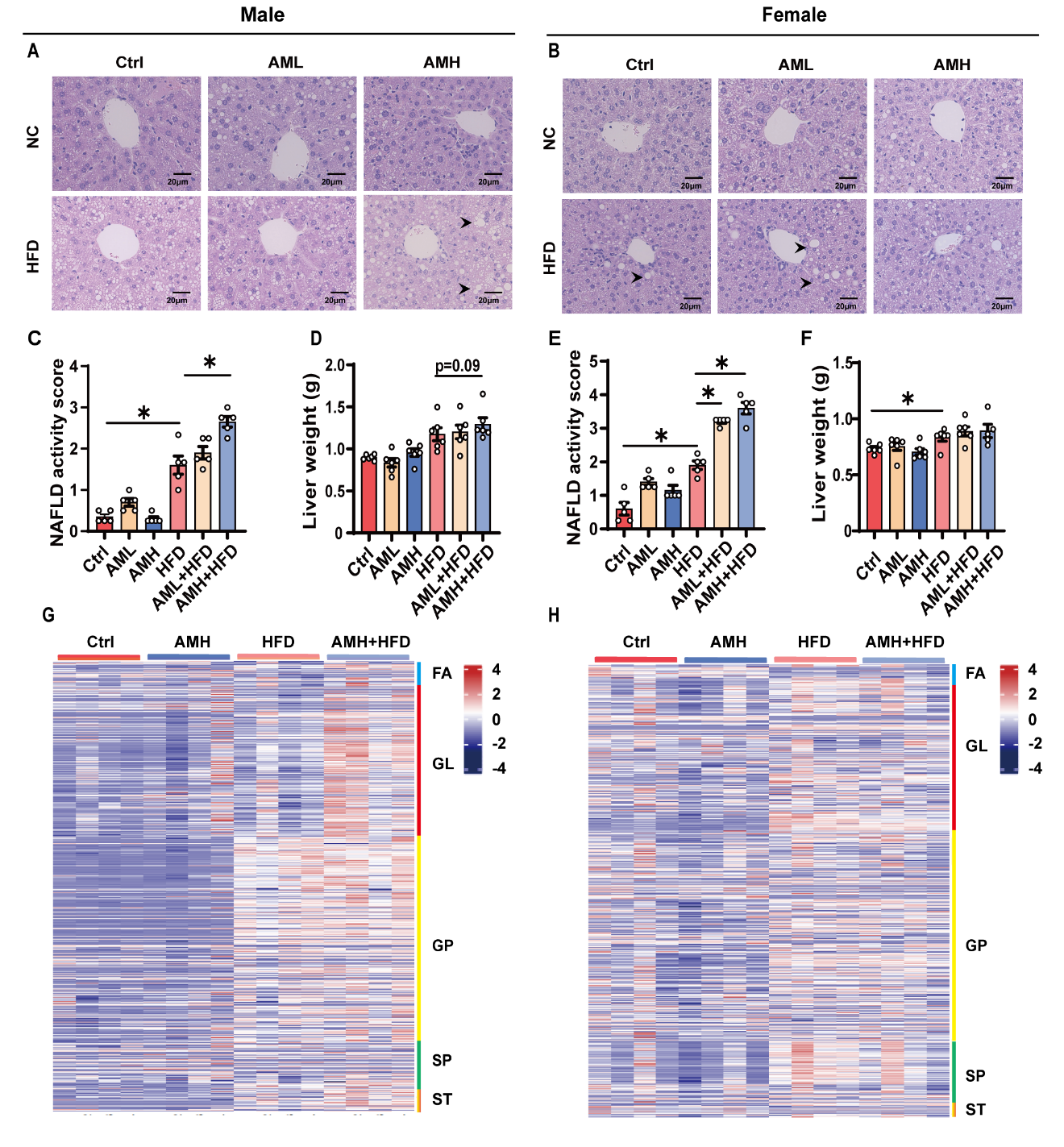
**

**Figure S2. Impact of perinatal AM exposure on lipid metabolism in HFD-fed adult offspring**

Maternal mice were administered with either NS, or low dose AM (AML, 20 mg/kg) or high dose AM (AMH, 200 mg/kg) by gavage from GD13 to PND7. All offspring were fed with normal chow (NC) from PND28 to PNW12. At PNW13, half were maintained on normal chow and half were fed with HFD for 6 weeks. At PNW19, liver tissues were collected. (A and B) Representative photomicrographs of hematoxylin and eosin (H&E) staining of liver tissues. Original magnification: ×400. (C and E) NAFLD activity score. (D and F) Liver weight. (G and H) A heatmap for subclass composition of total lipids. Data are presented as mean ± SEM, statistical significance was evaluated by two-sided one-way ANOVA with post hoc LSD tests. n = 4. *p < 0.05.

**
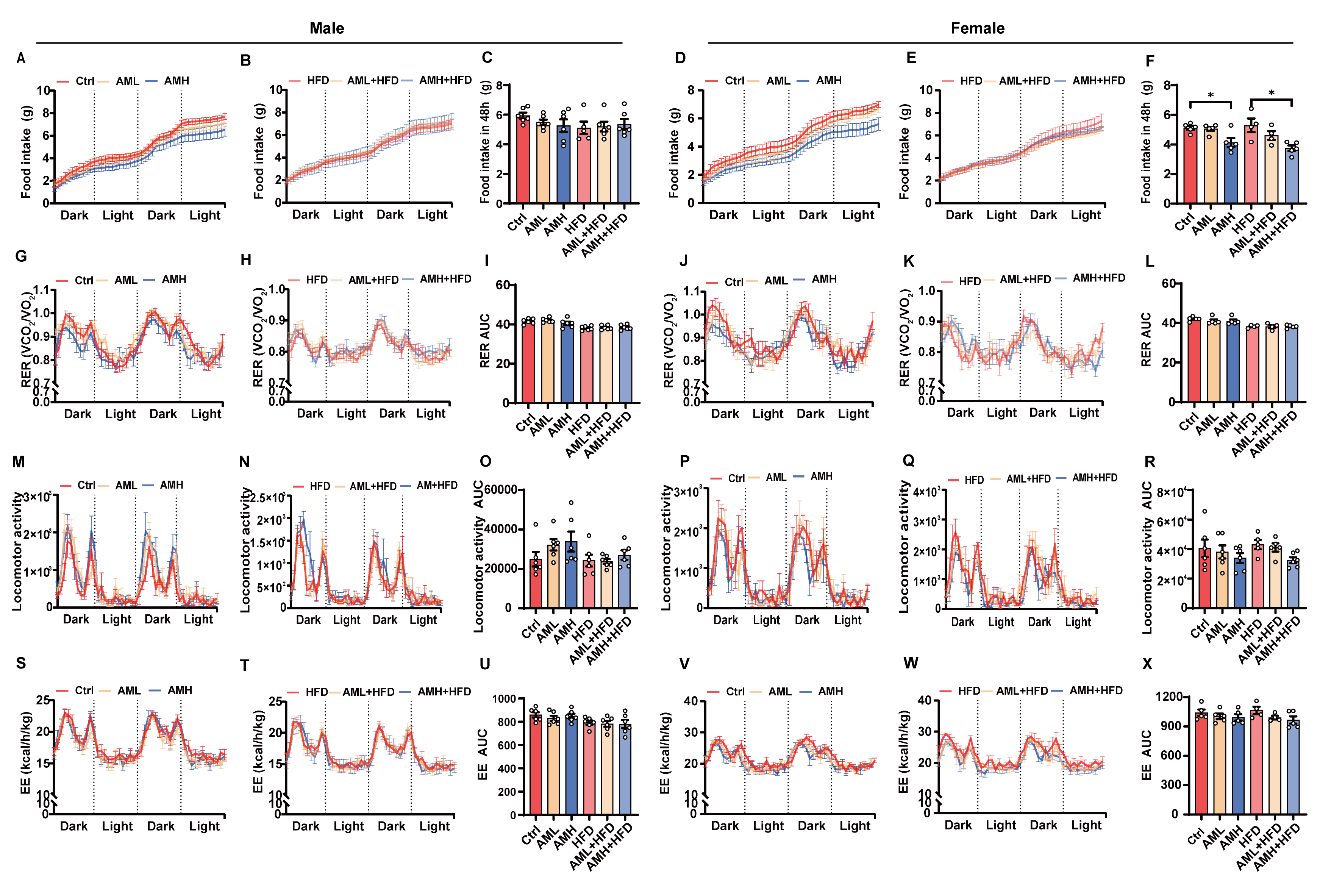
Figure S3. Impact of perinatal AM exposure on energy balance in HFD-fed adult offspring**

Maternal mice were administered with either NS, or low dose AM (AML, 20 mg/kg) or high dose AM (AMH, 200 mg/kg) by gavage from GD13 to PND7. All offspring were fed with normal chow (NC) from PND28 to PNW12. At PNW13, half were maintained on normal chow and half were fed with HFD for 6 weeks. On PNW17-18, energy metabolism was measured using the indirect caloric method. (A-F) Food intake within 48 h. (G, H, J and K) RER changes within 48 h. (I and L) AUC for RER was compared among different groups. (M, N, P and Q) Locomotor activity changes within 48 h. (O and R) AUC for locomotor activity was compared among different groups. (S, T, V and W) Energy expenditure changes within 48 h. (U and X) AUC for EE was compared among different groups. Abbreviations: EE, energy expenditure; RER, respiratory exchange ratio. Data are presented as mean ± SEM, statistical significance was evaluated by two-sided one-way ANOVA with post hoc LSD tests. n = 4-6. *p < 0.05.

**
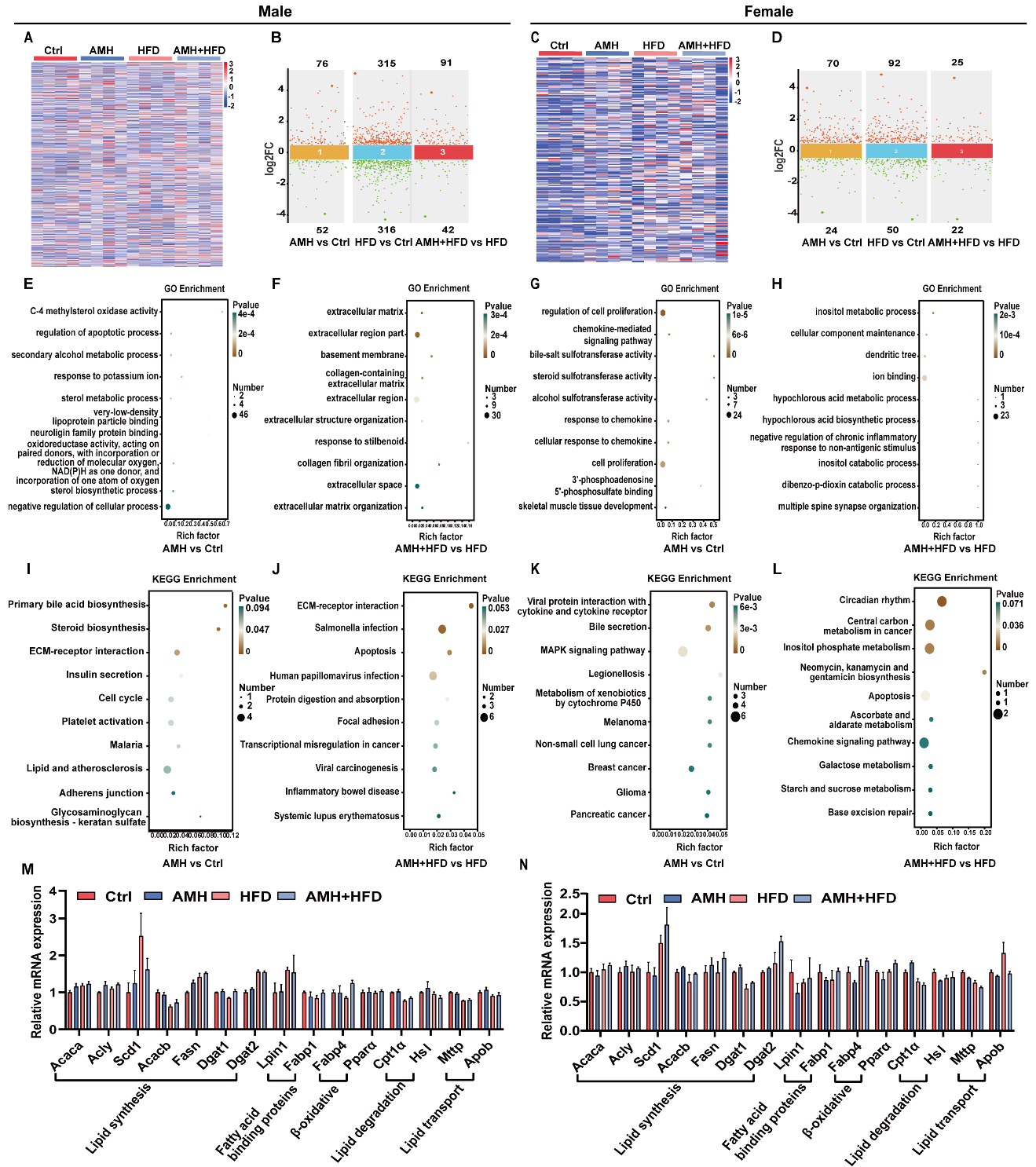
Figure S4. Impact of perinatal AM exposure on hepatic lipid metabolic genes in HFD-fed adult offspring**

Maternal mice were administered with either NS, or low dose AM (AML, 20 mg/kg) or high dose AM (AMH, 200 mg/kg) by gavage from GD13 to PND7. All offspring were fed with normal chow (NC) from PND28 to PNW12. At PNW13, half were maintained on normal chow and half were fed with HFD for 6 weeks. At PNW19, liver tissues were collected. Transcriptome was used to analyze gene expression. (A and C) Heat map for hepatic gene expression. (B and D) Dynamic differential scatter plots were used to compare the number of differential genes between two groups. (E-H) GO pathway was used to analyze the differentially expressed genes. (I-L) KEGG pathway was used to analyze the differentially expressed genes. (M-N) Hepatic lipid metabolic genes were measured using real-time RT-PCR. Data are presented as mean ± SEM, statistical significance was evaluated by two-sided one-way ANOVA with post hoc LSD tests.

n = 5-6. *p < 0.05.

**
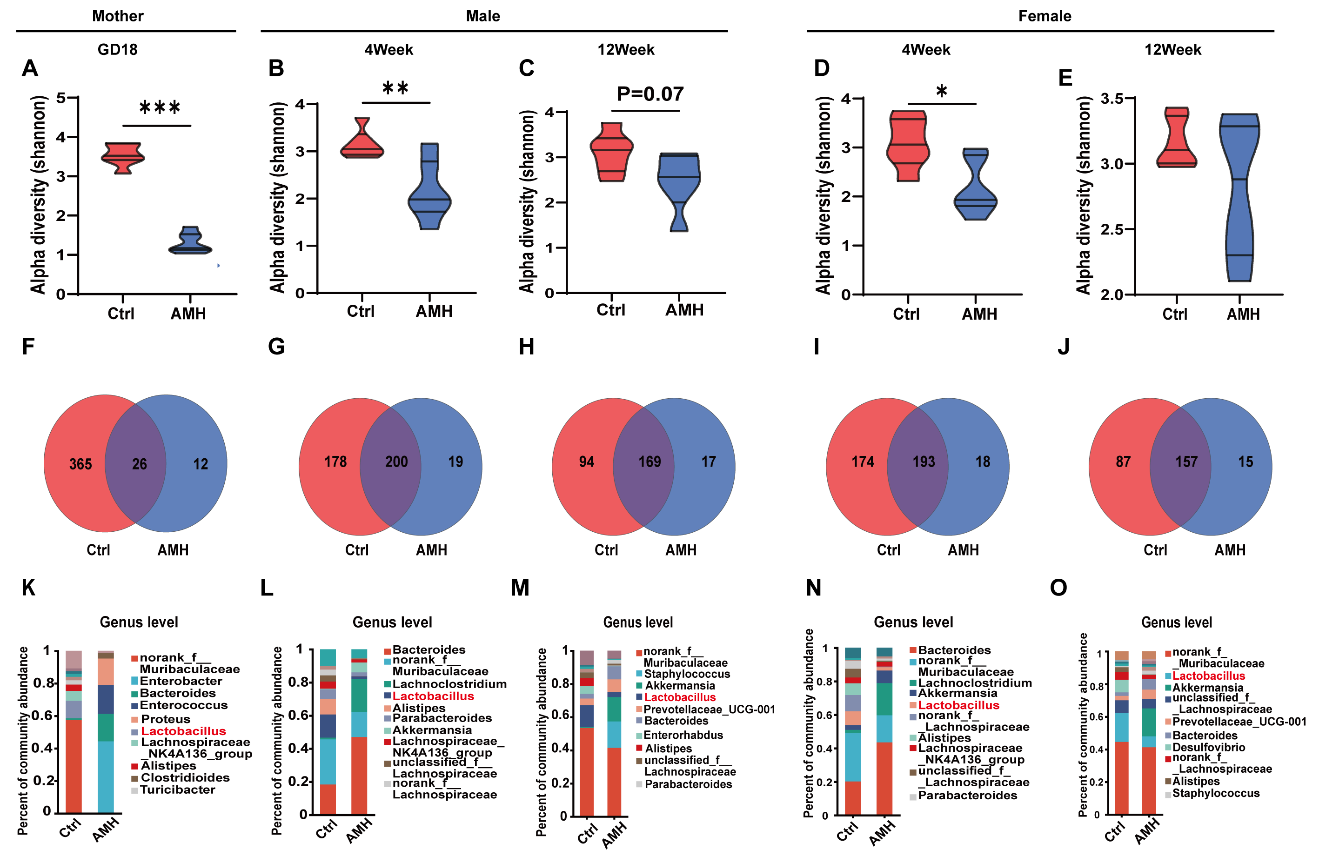
 Figure S5. Impacts of perinatal AM exposure on the richness and diversity of gut microbiota in maternal mice, weaned pups and adult offspring**

Maternal mice were administered either NS or AM (AMH, 200 mg/kg) by gavage from GD13 to PND7. All offspring were fed with normal chow (NC) from PND28 to PNW12. On GD18, maternal feces were collected. At PNW4 and at PNW12, Feces were collected from offspring. Gut microbiota was measured using 16S rRNA sequencing. (**A**-**E**) Shannon index of alpha diversity indicates diversity of bacterial community. (**F**-**J**) Venn diagrams showing number of species at the OTU level. (**K**-**L**) Community bar plot displays top 10 microbial compositions at genus level. All data were expressed as mean ± SEM, statistical significance was evaluated by two-tailed Student’s t-test. n = 6. *p < 0.05, **p < 0.01.

**
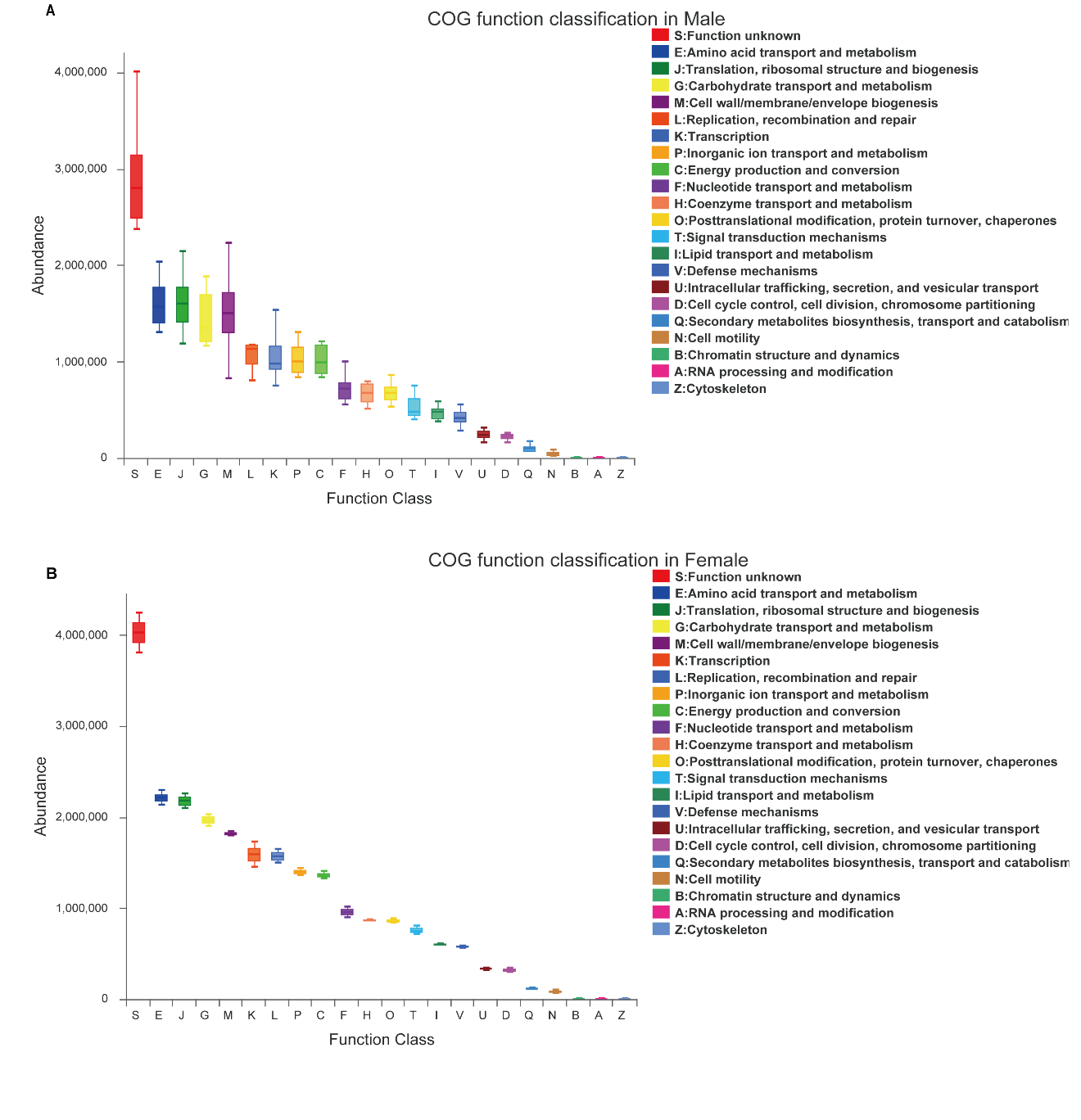
**

Figure S6. Functional prediction analysis of bacterial microbiota. PICRUSt2 combined with the eggNOG database to predict the function of bacterial microbiota in the feces of 12-week-old male and female offspring mice.

**
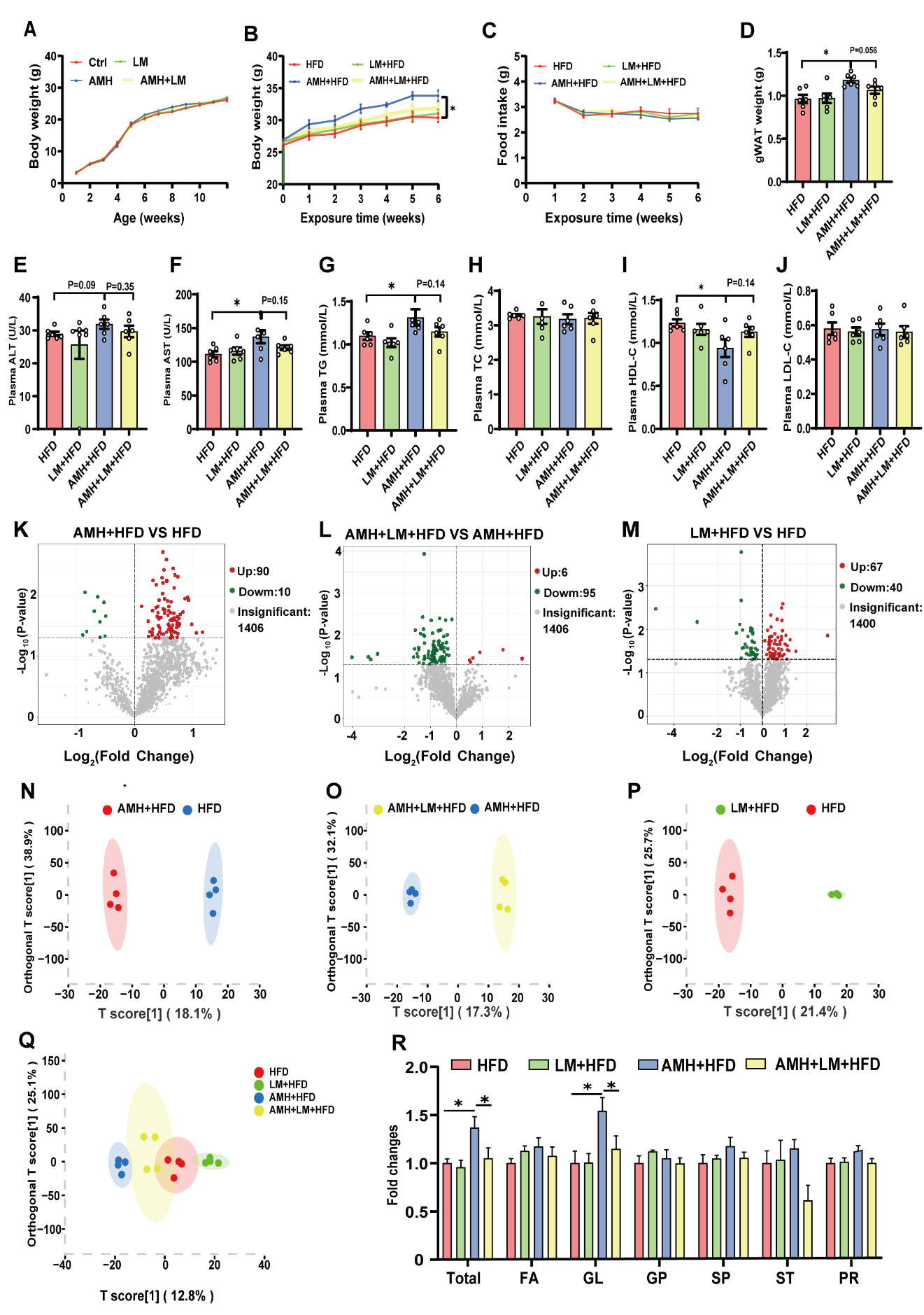
 Figure S7. Effects of supplementation with L. murinus on hepatic lipid profile in HFD-fed adult male offspring**

Maternal mice were administered with either NS or AM (AMH, 200 mg/kg) by gavage from GD13 to PND7. Half male pups were supplemented with L. murinus from PND8 to PND28. All male pups were fed with normal chow from PND28 to PNW12. At PNW13, half were maintained on normal chow and the other half were fed with HFD for 6 weeks. At PNW19, fat tissue, liver and blood plasma were collected. (A) Body weight changes before HFD feeding. (B) Body weight changes after HFD feeding. (C) Food intake after HFD feeding. (D) gWAT weight. (E-J) Plasma ALT, AST, TG, TC, HDL-C, and LDL-C were measured. (K and N) Volcano plot and OPLS-DA score plot represent the differential lipid metabolites between AMH-exposed HFD-fed and HFD-fed male offspring. (L and O) Volcano plot and OPLS-DA score plot represent the differential lipid metabolites between L. murinus-supplemented AMH-exposed HFD-fed and AMH-exposed HFD-fed male offspring. (M and P) Volcano plot and OPLS-DA score plot represent the differential lipid metabolites between L. murinus-supplemented HFD-fed and HFD-fed male offspring. (Q) OPLS-DA score plot was used to analyze difference among different groups. (R) Relative abundance of each subclass lipid was quantitatively analyzed. Abbreviations: FA, fatty acyls; GL, glycerolipids; GP, glycerophospholipids; gWAT, gonadal white adipose tissue; HFD, high-fat diet; PCA, principal component analysis; SP, sphingolipids; ST, sterol lipids. Data are presented as mean ± SEM, statistical significance was evaluated by two-sided one-way ANOVA with post hoc LSD tests. n = 4-5. *p < 0.05.

**
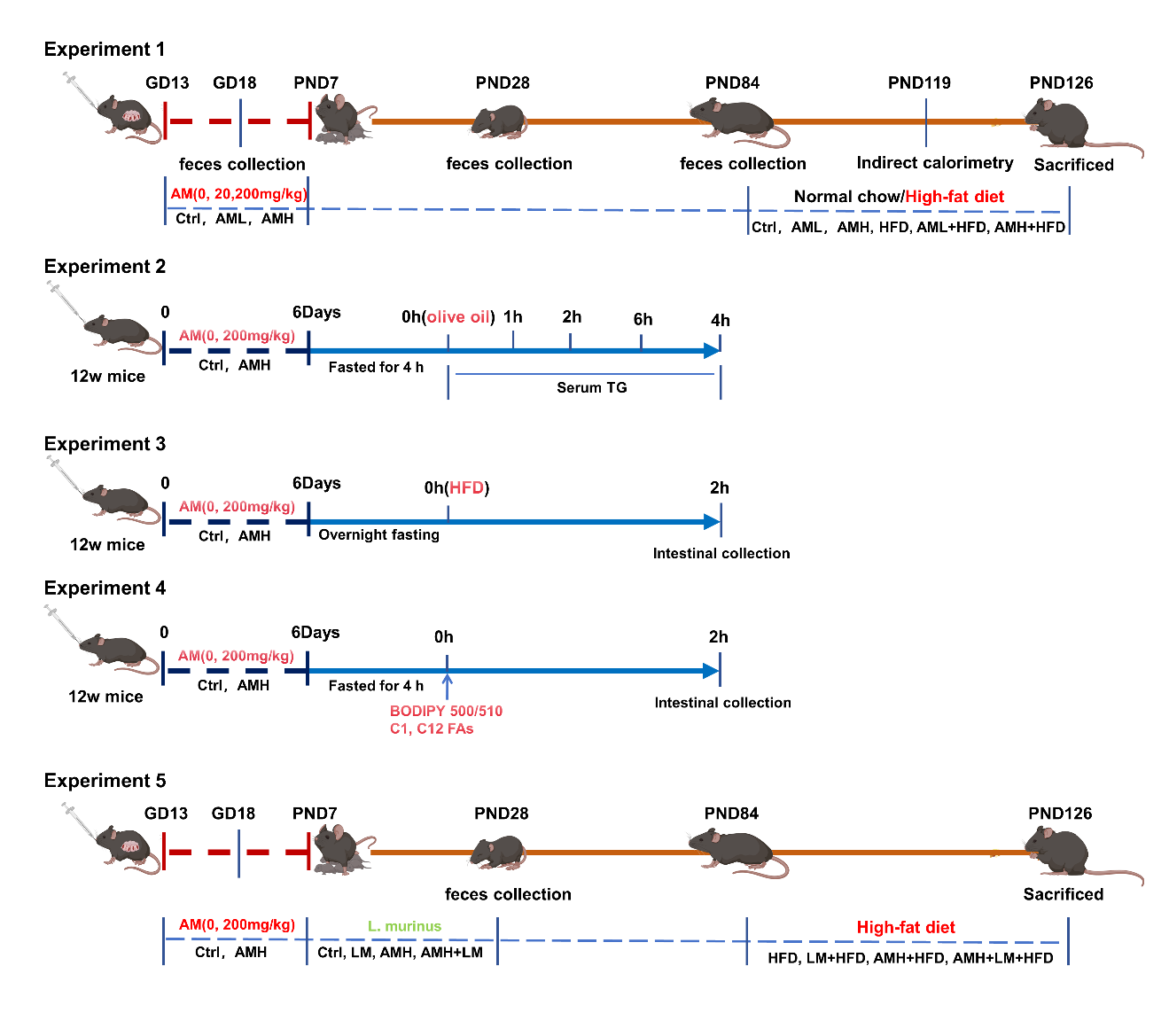
Figure S8 Experimental scheme for animals and treatments.**

Experiment 1, maternal mice were randomly assigned to three different groups. The maternal mice orally received different dose of AM (0, 20, 200 mg/kg) daily from GD13 to PND7. All pregnant mice gave birth naturally. On postnatal week (PNW)13, pups from different groups were divided into two subgroups: normal chow (NC) and high-fat diet (HFD) groups. In the NC group, pups were fed with normal chow. In the HFD group, pups were fed with a high-fat diet (HFD) for 6 weeks. Fecal samples were collected on gestational day 18 (GD18) of the mothers, and on postnatal days 28 (PND28) and 84 (PND84) of the offspring. Indirect calorimetry was measured on (PND119). Experiment 2, C57BL/6J mice were randomly divided into two groups and gavage with amoxicillin (200 mg/kg/d) for 6 days. After fasting for 4 h, baseline blood samples were collected from the mice via tail bleeding. Next, the mice were administered by gavage with olive oil. Blood was drawn by tail bleeding at 1 h, 2 h, 4 h and 6 h post oil bolus. Experiment 3, After six days of antibiotic exposure, animals are fasted overnight and refed 60% HFD for 2 h (n = 4 per group). Experiment 4, After six days of antibiotic exposure, mice were fasted for 4 h and administered with BODIPY 500/510 C1, C12 FAs, and olive oil for 2 h. Experiment 5, maternal mice were randomly assigned to two groups Ctrl and AMH. On PND8, pups from different groups were divided into two subgroups. with one half being administered Lactobacillus murinus by gavage. All mice were divided into four groups, Ctrl, LM, AMH and AMH+LM groups. On PNW13, all male offspring were fed a high-fat diet for 6 weeks.

**Supplementary references**

1. J. Hu, F. Deng, B. Zhao, et al. Lactobacillus murinus alleviate intestinal ischemia/reperfusion injury through promoting the release of interleukin-10 from M2 macrophages via Toll-like receptor 2 signaling. Microbiome. 10, 38 (2022).

2. C. L. Roth, C. T. Elfers, D. P. Figlewicz, et al. Vitamin D deficiency in obese rats exacerbates nonalcoholic fatty liver disease and increases hepatic resistin and Toll-like receptor activation. Hepatology 55, 1103–1111 (2012).

3. B. Wang, X. Rong, M. A. Duerr, et al. Intestinal Phospholipid Remodeling Is Required for Dietary-Lipid Uptake and Survival on a High-Fat Diet. Cell Metab. 23, 492-504 (2016).
